# Novel genes and alleles of the BTB/POZ protein family in *Oryza rufipogon*

**DOI:** 10.1038/s41598-023-41269-0

**Published:** 2023-09-19

**Authors:** Swarupa Nanda Mandal, Jacobo Sanchez, Rakesh Bhowmick, Oluwatobi R. Bello, Coenraad R. Van-Beek, Benildo G. de los Reyes

**Affiliations:** 1 Department of Plant and Soil Science, Texas Tech University, Lubbock, TX 79409 USA; 2 ICAR-Vivekananda Parvatiya Krishi Anusandhan Sansthan, Almora, Uttarakhand 263601 India

**Keywords:** Genetics, Plant sciences

## Abstract

The BTB/POZ family of proteins is widespread in plants and animals, playing important roles in development, growth, metabolism, and environmental responses. Although members of the expanded BTB/POZ gene family (*OsBTB*) have been identified in cultivated rice (*Oryza sativa*), their conservation, novelty, and potential applications for allele mining in *O. rufipogon*, the direct progenitor of *O. sativa* ssp. *japonica* and potential wide-introgression donor, are yet to be explored. This study describes an analysis of 110 BTB/POZ encoding gene loci (*OrBTB*) across the genome of *O. rufipogon* as outcomes of tandem duplication events. Phylogenetic grouping of duplicated *OrBTB* genes was supported by the analysis of gene sequences and protein domain architecture, shedding some light on their evolution and functional divergence. The *O. rufipogon* genome encodes nine novel BTB/POZ genes with orthologs in its distant cousins in the family Poaceae (*Sorghum bicolor*, *Brachypodium distachyon*), but such orthologs appeared to have been lost in its domesticated descendant, *O. sativa* ssp. *japonica*. Comparative sequence analysis and structure comparisons of novel *OrBTB* genes revealed that diverged upstream regulatory sequences and regulon restructuring are the key features of the evolution of this large gene family. Novel genes from the wild progenitor serve as a reservoir of potential new alleles that can bring novel functions to cultivars when introgressed by wide hybridization. This study establishes a foundation for hypothesis-driven functional genomic studies and their applications for widening the genetic base of rice cultivars through the introgression of novel genes or alleles from the exotic gene pool.

## Introduction

The genus *Oryza* is comprised of two cultivated species of Asian (*Oryza sativa;* 2n = 24 = AA) and African (*Oryza glaberrima*; 2n = 24 = AA) origins, and twenty-five wild species with either diploid or tetraploid genomes (2n = 24 = AA, BB, CC, EE, FF, GG; 2n = 48 = BBCC, CCDD, HHJJ, HHKK, KKLL)^1,2^. It is well established that both the indica (tropical) and japonica (temperate) subspecies of *O. sativa* were domesticated from the wild species *O. rufipogon* (2n = 24 = AA) approximately 9000 years ago^3–5^. Compared to their wild progenitors, domesticated rices have undergone significant phenotypic and physiological changes due to intensive selection, as evident from the relatively limited variability across the cultivated germplasm^6–8^. Such genetic bottleneck effect has become a major limitation to breeding new cultivars for enhanced resilience to the drastically changing ecology triggered by climate change and natural resource deterioration^9,10^. The biodiversity and bioclimatic ranges across the wild germplasm, particularly in the progenitor *O. rufipogon*, represent a wider potential and rich reservoir of novel genes and/or alleles that have been left behind by domestication. This accessible novel gene pool is a critical element of the new breeding paradigm for widening the genetic base of future cultivars with enhanced capacity to maintain yield under sub-optimal environments^5,11–19^.

The biochemical, physiological, and developmental plasticity of plants in response to drastic environmental changes can either be adaptive or non-adaptive^20^. Adaptive or not, these responses are configured by intricate molecular changes, initiated by the perception of extrinsic or intrinsic signals and culminating with altered biochemical, physiological, or developmental status at the cellular, organ, or whole-plant levels^21,22^. Adaptive responses lead to effective defense or avoidance mechanisms by virtue of well-orchestrated biochemical networks facilitated by regulatory genes that act either at the level of protein modification, turnover, or de novo synthesis, leading to either activation or repression of the transcription of genes that execute cellular adjustment and defense-related processes^23,24^.

Also important to the fidelity of cellular adaptive responses are the molecular ‘*fine-tuners*’ that integrate the various aspects of regulation. Among the many known examples of fine-tuners, the BTB (*Broad-complex, Tramtrack and Bric a brac*) class of proteins, also known as POZ (*Pox virus and Zinc finger*) proteins, play important roles in the integration and modulation of molecular cascades that facilitate adjustment of plant growth and development under stress^25–30^. First discovered in *Drosophila*, proteins containing the BTB/POZ domains are evolutionarily conserved across metazoans and plants, and they have been associated with diverse functions primarily as scaffolding proteins^25,31^. Other members of this protein family function in the regulation of transcription, chromatin remodelling, cytoskeleton dynamics, ion channel formation, and ubiquitination of other regulatory proteins^32–39^.

The BTB/POZ domain has been established as a substrate receptor for Cullin 3 (CUL3)-based E3 ligases^25^. The canonical feature of the BTB/POZ proteins is a tightly entwined homodimer with a core made-up of five α-helices, with A1/2 and A4/5 forming two α-helical hairpins and three β-strands forming a β-sheet^39,40^. These structures and the elements that form part of an E3 ubiquitin ligase complex enable inter-molecular interaction of the BTB/POZ proteins with other proteins to execute their regulatory functions^41^. In addition to the highly conserved BTB/POZ domain, other sub-classes may also contain one or more additional domains, including the NPR1/NIM1(NON-EXPRESSOR OF PATHOGENESIS-RELATED PROTEIN1/NON-INDUCIBLE IMMUNITY1), CaMBD (calmodulin-binding domain), NPH3 (NON-PHOTOTROPIC HYPOCOTYL 3), Armadillo/beta-catenin-like repeat, signal peptide peptidase, kelch motifs, tetratricopeptide repeat (TPR), MATH (MEPRIN AND TUMOUR NECROSIS FACTOR RECEPTOR-ASSOCIATED FACTOR HOMOLOGY), ANK (Ankyrin repeats), TAZ (transcriptional adapter zinc finger), Skp1 (S-phase kinase-associated protein), DUF (domain of unknown function) and the C2-like domain^29,39,42^. These domains are important for a wide range of molecular functions, contributing to the functional diversity across the protein family^43^.

In general, the BTB/POZ domain consists of approximately 95 amino acid residues, with various degrees of sequence and length variation between orthologs and paralogs. These variations imply potential significance in specifying diverse molecular, biochemical, and biological functions across plants and animals^39^. Furthermore, studies comparing the orthologs of BTB/POZ proteins across monocot and dicot plants have shown evidence that the protein family was created through a “*rapid birth-and-death evolution*” mechanism, which enabled different members of the family to bind to a wide range of substrates^25,43–45^.

Genome-wide analysis of the BTB/POZ gene family in diverse species of plants, including Arabidopsis, rice, tomato, cucumber, and sugarbeet, reiterated their diverse biological functions^28,46–48^. Members have been shown to be involved in the regulation of inflorescence architecture and branching, specification of spindle length and nuclear identity, gametophyte development, and seed germination^26,27,49–51^. Others have been implicated with various types of environmental responses, including defenses against pathogens, herbivores, and parasites, and responses to nutrient depletion, toxic heavy metals, and many other types of stressors^19,30,52–58^.

The involvement of the BTB/POZ protein family in the regulation of plant responses to environmental stressors suggests their potential applications in stress tolerance engineering. In rice, one of the major approaches is the introgression of novel genes or alleles from the exotic gene pool into cultivars. As direct progenitor of the cultivated rice (*O. sativa*), the wild species *O. rufipogon* has been shown as a rich source of such novel genes/alleles that are accessible for the diversification of the genetic base of *O. sativa* given the ease of sexual hybridization between the two species. In this study, we examined the *O. rufipogon* genome as a potential donor of exotic genes or alleles encoding BTB/POZ proteins. Here we report a systematic survey and phylogenetic characterization of all the BTB/POZ protein-encoding genes of *O. rufipogon* and their implications to the structural, functional, and evolutionary dynamics in the direct wild progenitor of the Asian cultivated rice *O. sativa* ssp. *japonica*. We discuss the potential implications of the diversity across the gene family in mining for novel alleles from *O. rufipogon* by direct comparison with the cultivated rice and other distant lineages in the monocot branch.

## Materials and methods

### Identification of BTB/POZ genes in *O. rufipogon* genome

The most recent versions of *O. rufipogon* genome^2^, peptide, and cDNA sequences (CDS) and their annotation were obtained from Ensembl databases (https://plants.ensembl.org/Oryza_rufipogon/Info/Index). The HMM (Hidden Markov Model) profiles of the BTB/POZ domain were obtained from the Pfam database (http://www.pfam.xfam.org/). Initial identification of the BTB/POZ domain-containing genes in the genome and protein sequence databases was performed by scanning the entire HMM profiles with the conserved sequences of the BTB/POZ domain (PF00651) as a query at a threshold E-value of 1.0. After establishing an initial shortlist of candidates based on HMM profiles, the putative BTB/POZ gene sequences were subjected to Pfam and CDS searches to validate the BTB/POZ domains. Because the HMM search could fail in identifying truncated BTB/POZ domains, an additional BlastP search was also performed to reveal the signatures of partial domains among the putative BTB/POZ-encoding genes. For the BlastP search, the confirmed BTB/POZ-encoding genes identified by HMM profiles were used as queries against the entire protein sequence database of *O. rufipogon* at a threshold E-value of 10^−5^. Genes identified using the two approaches were merged to get the maximum number of candidate BTB/POZ-encoding genes.

### In silico characterization of BTB/POZ genes and their encoded proteins

To visualize the distribution of BTB/POZ-encoding genes across the annotated sequences of each *O. rufipogon* chromosome, the GFF3 annotation file was parsed to extract the genomic location of each gene locus. Graphical presentation of the physical position of BTB/POZ genes was done by MapChart Tool at default parameters^59^. The intron–exon architecture was mapped using the GSDS2.0 server (http://gsds.gao-lab.org/) by comparing the genomic sequences with the corresponding CDS for each gene locus.

The BTB/POZ proteins encoded by each gene locus were characterized by predicting their physico-chemical properties, sub-cellular location, and transmembrane domains. Physico-chemical properties were predicted based on amino acid composition, dipeptide composition, partitioned amino acid composition, molecular weight, and isoelectric point (pI) using publicly available analytical tools such as CELLO v.2.5 (http://cello.life.nctu.edu.tw/), Isoelectric Point Calculator (http://isoelectric.org/), and ExPasy Proteomics (https://web.expasy.org/compute_pi/) at default parameters. Promoters of BTB/POZ genes were delineated within 2000 bp upstream of the predicted transcription start site (TSS) using the “blastdbcmd” of stand-alone BLAST (https://ftp.ncbi.nlm.nih.gov/blast/executables/LATEST/). Analyses of conserved protein sequence motifs and promoter sequence motifs were performed with the MEME suite (https://meme-suite.org/meme/). Protein sequence motifs were examined at the minimum and maximum motif width of 6-aa and 20-aa, respectively, for a maximum of 10 motifs using the zoops model^60^. Nucleotide sequence motifs identified across the upstream regions of each promoter were translated into putative cis-elements based on homology in the PlantCare database (http://www.bioinformatics.psb.ugent.be/webtools/plantcare/html/)^61^. The distribution of putative cis-elements along the promoter sequences was presented graphically using the TBtools software^62^. Amino acid sequence alignment between *O. rufipogon*, *O. sativa japonica*, *Brachypodium distachyon* and *Sorghum bicolor* orthologs and paralogs was through the Clustal Omega (https://www.ebi.ac.uk/Tools/msa/clustalo/) and CLUSTALW (http://www.ebi.ac.uk/clustalw/) with default parameters, visualized with MView analysis tools (https://www.ebi.ac.uk/Tools/msa/mview/).

### Phylogenetic analysis

Coding sequences of BTB/POZ protein-encoding genes were first aligned using ClustalW (https://www.ebi.ac.uk/Tools/msa/clustalo/)^63^*.* Multiple sequence alignment files were imported to MEGA7 and used for phylogenetic analysis through the Neighbor-Joining approach^64,65^. Analysis of the evolutionary history of the taxon was performed by bootstrap consensus tree estimated from 1000 repetitions (https://www.megasoftware.net/)^66^, with no fewer than 50% replication of branch partitions. Evolutionary distances were calculated using the p-distance method and expressed in amino acid changes per location across a total of 110 amino acid sequences^65^. Unclear locations were discarded for each pair of comparison. In total, 3412 positions were included in the final dataset.

### Analysis of homology and gene duplication

Analysis of gene duplication events was performed with the MCScanX toolkit. Paralogs in *O. rufipogon* were identified by the duplicate gene classifier program of the MCScanX toolkit at default parameters. Circular synteny plots depicting duplicated genes were visualised with the TBtools^62^. Interspecies syntenic relationships between *O. rufipogon* and *O. sativa* japonica (IRGSP-1.0), *Arabidopsis thaliana* (TAIR10), *Brachypodium distachyon* (Brachypodium_distachyon_v3.0), and *Sorghum bicolor* (Sorghum_bicolor_NCBIv3) were established using the dual synteny package of TBtools at threshold E-value^62^ of < 1 × 10^−10^. The Ka–Ks calculator of TBtools was used to estimate the Ka/Ks value of duplicated gene pairs. Based on the approximate substitution rate with r = 6.5 × 10^−9^ per site, the divergence time (T) was calculated by T = Ks/2r × 10^−6^ million years ago (MYA)^67^.

### Analysis of gene ontology, miRNA target sites, and protein–protein interaction

Plant miRNA-targeted gene prediction was performed with psRNATarget (https://www.zhaolab.org/psRNATarget/)^68^. Gene Ontology (GO) analysis was through the shinny_go v07.41 (http://bioinformatics.sdstate.edu/go/)^69^. The BTB/POZ protein sequences were used to predict and visualize potential Protein–Protein Interaction (PPI) networks using the STRING analytical tools (https://string-db.org/)^70^.

### In silico expression profiling of BTP/POZ genes

Spatio-temporal transcriptomic datasets of *O. rufipogon* were obtained from the Short Read Archive aided by the SRA Toolkit (i.e., *accession SRP151515 for tissues and developmental stages; accession SRP198462 for iron deficiency; accession SRP063832 for salinity stress; accession SRP251791 for cold stress*). Sequence reads were filtered and trimmed using the Trimmomatic tools^71^, and aligned to the *O. rufipogon* reference genome with Bowtie2 at default parameters^72^. The Kallisto analysis tools (v0.46.1) were used to quantify transcript abundances as transcripts per million reads (TPM)^73^. Averaged TPM were log-transformed (Log2X) to create heatmaps with TBtools software^62^.

### Analysis of BTB/POZ gene expression

*O. rufipogon* accession IRGC105491 used in all experiments was obtained from the collection of Prof. Susan McCouch, Cornell University, USA. Dehulled seeds were surface sterilized with 5% (w/v) sodium hypochlorite and germinated at 30 °C in a growth chamber. Seedlings were established in seed trays with standard vermiculite potting mix and acclimated in half-strength Yoshida nutrient media for 21 days. The Yoshida media was comprised of 0.4 mM NH_4_NO_3_, 10 mM KNO_3_, 2 mM CaNO_3_, 2 mM MgSO_4_, 0.1 mM KH_2_PO_4_, 1.5 mM CaCl_2,_ and other micronutrients comprised of 0.1 mM Fe-EDTA, 12.5 µM H_3_BO_3_, 2 µM MnCl_2_, 3 µM ZnSO_4_, 0.5 µM CuSO_4_, 0.1 µM Na_2_MoO_3_, 0.1 µM NiSO_4_ and 25 µM KCl with a pH of 6.0.

Healthy seedlings were subjected to phosphate deficiency stress in optimal Yoshida nutrient media in the absence of KH_2_PO_4_. Nitrogen deficiency stress treatments were in the same optimal media in the absence of NH_4_NO_3_, KNO_3_, CaNO_3_. Iron toxicity stress experiments were conducted on 21-day-old seedlings under optimal media amended with 2 × FeSO_4_·7H_2_O (0.8 g/L). For salinity stress, plants were first established under optimal hydroponic media supplemented with 0.4 g/L FeSO_4_·7H_2_O for 8 weeks and then transferred to a nutrient solution salinized with NaCl^74^ at EC = 12 dS m^−1^. Nutrient and salinity stress experiments were performed under greenhouse conditions with 30–35 °C day/24–26 °C night temperature cycle, 20–30% RH, and 14-h photoperiod. For heat stress experiments, 5-day-old seedlings were first established in optimal Yoshida nutrient media. After 14 days, seedlings were exposed to an elevated temperature of 42 °C day/28 °C night cycle with 30% RH and 14-h photoperiod in a growth chamber. Temporal sampling of plant tissues under control and stress conditions was performed in triplicates. For heat and salinity stresses, samples were collected at 0 h (control) and after 24, 48, and 72 h of stress. Tissue samples were collected after 14 days in the phosphate and nitrogen deficiency experiments and also in the iron toxicity experiments.

### Total RNA extraction and qRT-PCR analysis

The Spectrum Plant Total RNA Kit (Sigma, St. Louis, MO) was used to extract total RNA according to the manufacturer's instructions. RNA integrity was evaluated by agarose gel electrophoresis, and the concentration was measured by NanoDrop™ Lite (ThermoFisher Scientific, USA). Synthesis of cDNA was performed with 500 ng total RNA using the iScript cDNA synthesis kit according to the manufacturer’s instructions (Bio-Rad, Hercules, CA). Gene-specific primers for qRT-PCR were designed using Primer3 (v. 0.4.0), and their specificities to the target genes were confirmed by Primer-BLAST (Supplementary Table 1). The qRT-PCR assay was performed using the Bio-Rad SsoAdvanced™—Universal SYBR^®^ Green Supermix in the Bio-Rad CFX Connect—Real-Time PCR System with three biological and two technical replicates. The qRT-PCR cycling was performed at 95 °C for 30 min, followed by 40 cycles of 95 °C for 10 s (denaturation) and 60 °C for 30 s (annealing and extension). Relative changes in transcript abundance were calculated by the 2^−ΔΔCt^ method^75^. Each treatment was compared with the control using Dunnett’s *t*-test (p < 0.05).

## Results

### Distribution and phylogeny of BTB/POZ genes in O. rufipogon genome

The HMM profile and BlastP outputs yielded a total of 128 preliminary hits, which were confirmed by Pfam searches to verify the occurrence of *bona fide* BTB/POZ domains. Of the total preliminary hits, 110 genes were determined to contain *bona fide* BTB/POZ domains, while the rest were potential random homologies. Because the HMM profile search often misidentifies truncated domains, the 110 putative BTB/POZ protein-encoding gene loci were used as queries for another iteration of BlastP search across the *O. rufipogon* protein database. This extra iteration did not reveal additional gene copies, thus 110 putative BTB/POZ protein-encoding genes of *O. rufipogon* with genomic loci identifier (*OrBTB1* to *OrBTB110*) comprised the final gene set from all chromosomes (Supplementary Table 2). In silico analysis of the physico-chemical attributes of putative BTB/POZ proteins revealed diverse properties across the gene family with molecular weights ranging from 18.49 kDa to 281.12 kDa and an average of 58.76 kDa (Supplementary Table 2), and isoelectric points (pI) ranging from 4.27 to 10.55 and average of 6.19.

**Figure 1:**
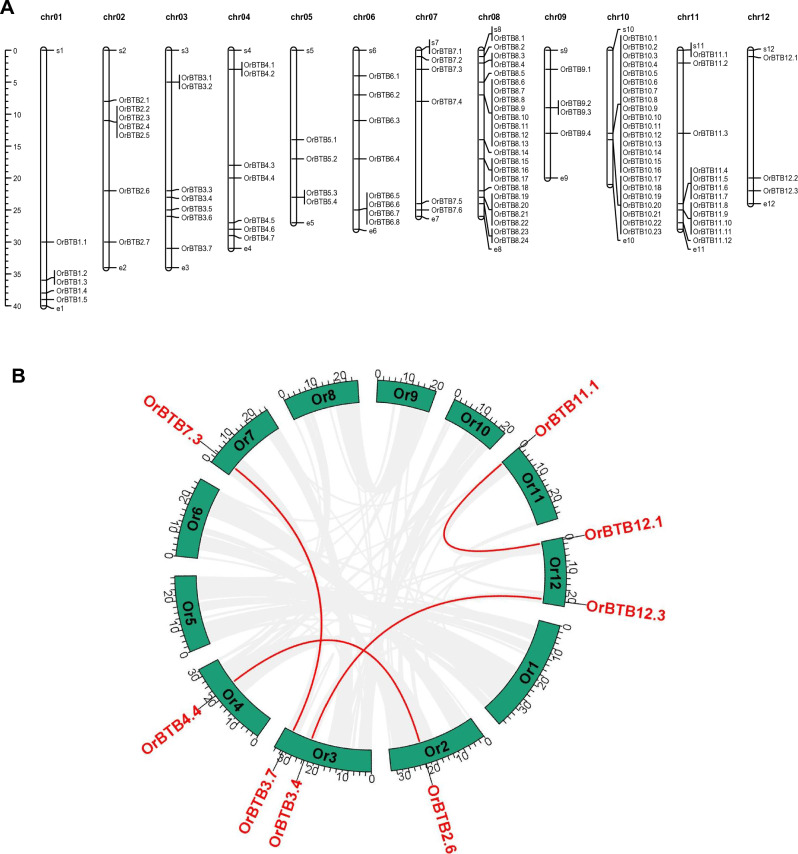
(**A**) Graphical presentation of the physical location of *OrBTB* gene copies across the 12 chromosomes of *O. rufipogon* generated by MapChart Tool. The left bar indicates the scale of chromosome length in a million base pairs (Mbp). (**B**) Circos plot showing the segmentally duplicated *OrBTB* gene pairs in *O. rufipogon* genome. Green bars represent different chromosomes of *O. rufipogon*. Red-colored lines represent gene pairs that are putative products of segmental duplication. Grey lines in the background represent conserved synteny blocks. The figure was generated using advance circos program of TBtools software.

BTB/POZ gene loci were distributed across all 12 chromosomes of *O. rufipogon* (Fig. 1A). Chromosomes-08 and -10 contained the largest number of copies with 24 and 23 loci, respectively, while chromosome-12 contained the lowest number with only three loci. Evident in the chromosomes with the largest number of loci, BTB/POZ genes occurred in small to large tandem arrays, indicating their origins through tandem duplication. For instance, a large cluster of 23 loci occurred in a region of chromosome-10 with coordinates of 13.12 Mb and 13.74 Mb (total cluster size of 0.62 Mb). Smaller tandem arrays were located on chromosomes-11 and -02, with seven members (coordinates = 24.04–26.62 Mb), and four members (coordinates = 11.25–11.33 Mb), respectively. Two small tandem arrays were located on chromosome-08, with eight (coordinates = 6.79–6.95 Mb) and six (coordinates = 6.79–6.95 Mb) members, respectively. Prediction of subcellular localization showed that most OrBTB proteins were localized in the cytoplasm (30 genes), chloroplast (29 genes), nucleus (23 genes), plasma membrane (17 genes), mitochondria (8 genes), and extracellular space (3 genes) (Supplementary Table 2; Supplementary Fig. 1).

**Figure 2:**
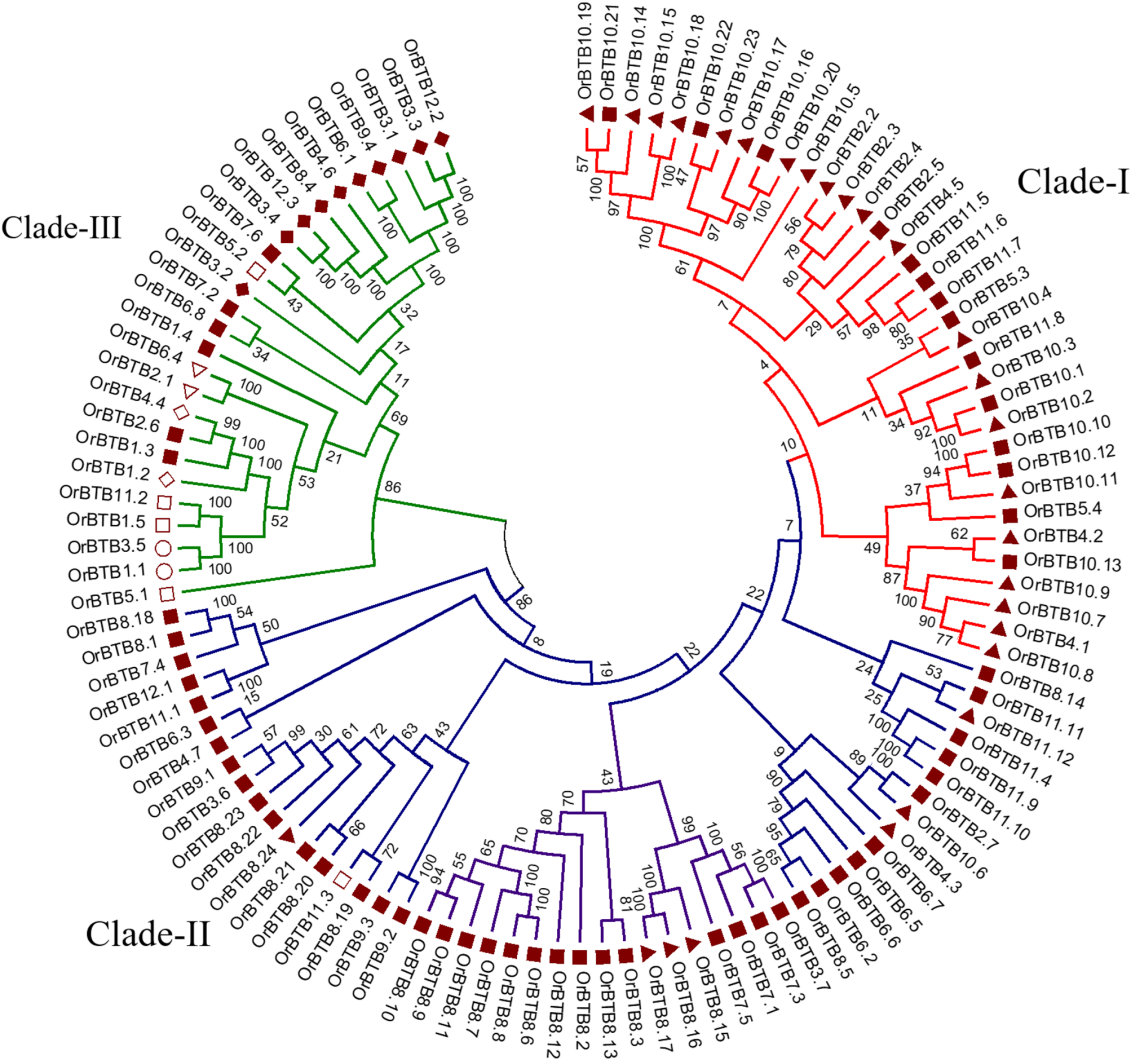
Neighbor-joining phylogenetic tree of 110 *OrBTB* gene copies encoded in *O. rufipogon* genome. Different groups and clades are distinguished by the color of branches. Conserved domains in the map of each BTB/POZ protein are represented as a solid triangle (MATH), solid diamond (NPH3), hollow circle (NPR1), solid square (BTB/POZ only), hollow triangle (BACK), hollow diamond (zf-TAZ), and hollow square (others). Figure was generated by Mega v7 software.

The unrooted tree constructed by the Neighbor-Joining method showed that the 110 BTB/POZ-encoding genes of *O. rufipogon* formed three phylogenetically distinct sub-groups (Fig. 2). The largest group (clade-2) was comprised of 49 members, of which the majority (88% = 43 genes) had only the BTB/POZ domain, while a smaller minority of its members contained other additional domains such as MATH (5 genes) and PA/peptidase (1 gene). Eight of the BTB/POZ genes in clade-2 were part of a small tandem array on chromosome-08 (*OrBTB8.6/ORUFI08G07460, OrBTB8.7/ORUFI08G07490, OrBTB8.8/ORUFI08G07520, OrBTB8.9/ORUFI08G07540, OrBTB8.10/ORUFI08G07550, OrBTB8.11/ORUFI08G07560, OrBTB8.12/ORUFI08G07610, OrBTB8.13/ORUFI08G07650*) along the 6.7–6.9 Mb genomic coordinates. As the general structures of these genes were not significantly similar to each other, they appeared to have been created through tandem duplication events followed by structural and functional diversification. Another tandem array on chromosome-08 comprised of *OrBTB8.19/ORUFI08G22900, OrBTB8.20/ORUFI08G22910, OrBTB8.21/ORUFI08G22920, OrBTB8.22/ORUFI08G22930, OrBTB8.23/ORUFI08G23440* and *OrBTB8.24/ORUFI08G23450* in genome coordinates 23.2–23.6 Mb clustered in the same sub-group in clade-2.

Clade-1 was comprised of 35 members, of which 21 also contained the MATH domain. Surprisingly, out of the 35 members in this clade, 22 were located in a tandem array on chromosome-10 (13.1–13.7 Mb), signifying that members of this sub-group were the outcomes of the largest duplication event in this protein family. The smallest clade (clade-3) consisted of 26 members, of which ten genes had an NPH3 domain *(OrBTB 3.1/ORUFI03G07610, OrBTB 3.2/ORUFI03G07690, OrBTB 3.3/ORUFI03G26720, OrBTB 3.4/ORUFI03G28320, OrBTB4.6/ORUFI04G28350, OrBTB6.1/ORUFI06G05610, OrBTB 8.4/ORUFI08G02180, OrBTB 9.4/ORUFI09G11290, OrBTB12.2/ORUFI12G19040, OrBTB12.2/ORUFI12G19040*), two had a BACK domain (*OrBTB2.1/ORUFI02G11310, OrBTB6.4/ORUFI06G17340*), two had an NPR1 domain (*OrBTB1.1/ORUFI01G35500, OrBTB3.5/ORUFI03G29880*), and two had a TAZ domain (*OrBTB1.2/ORUFI01G43500, OrBTB4.4/ORUFI04G17810*). It appeared that this small clade was comprised of genes with much more diverse functions.

### Structure of *O. rufipogon* BTB/POZ genes and their protein products

**Figure 3:**
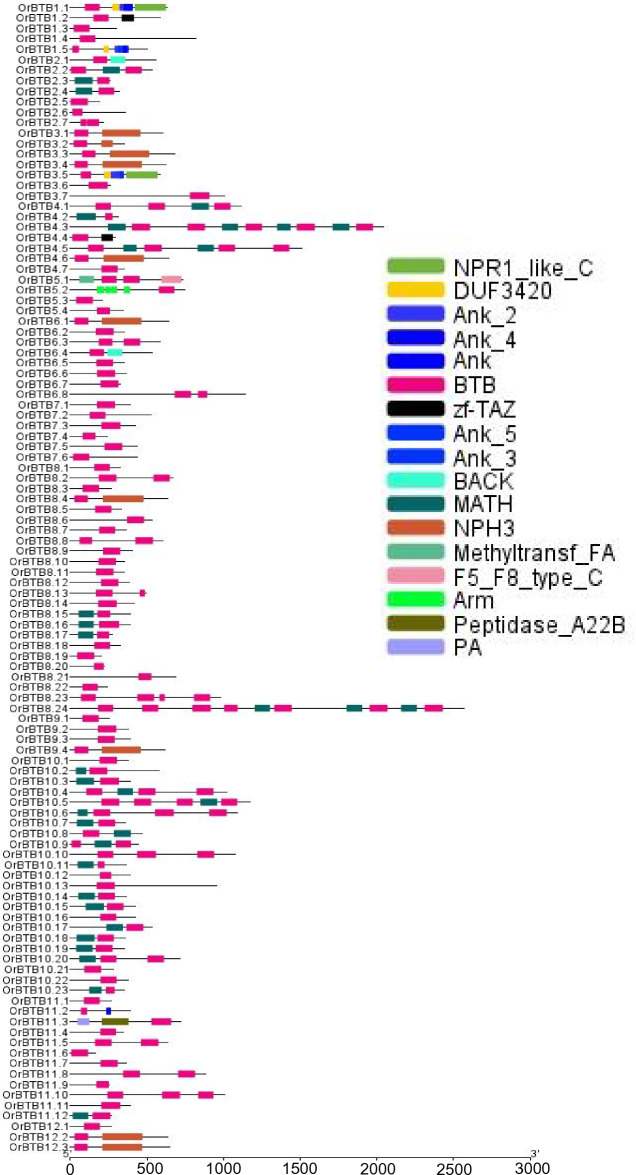
Comparison of the domain architecture of *OrBTB* proteins encoded by the 110 gene family members in *O. rufipogon*. Conserved domains are color-coded. Domains were identified using pfam server. Domains were represented using software TBtool v1.068.

The majority (87 of 110) of the BTB/POZ proteins contained only one copy of the BTB/POZ domain, while the other members had multiple copies (2–7) (Fig. 3). The MATH domain was the most common combination with the BTB/POZ domain, with a total of 34 members (out of 110) having such a combination. Of these, 25 members had one MATH domain and three others had more than two copies of the MATH domain. Some BTB/POZ members combined with other functional domains such as Ank (13 members), NPH3 (10 members), DUF3420 (3 members), zf-TAZ (2 members), BACK (2 members), NPR1_like (2 members), Arm (1 member), F5_F8_ty (1 member), Methyltr (1 member), PA (1 member), and Peptidase (1 member). The combinatorial occurrence of these other domains suggests that the BTP/POZ gene family of *O. rufipogon* may have also undergone the process of domain shuffling.

**Figure 4:**
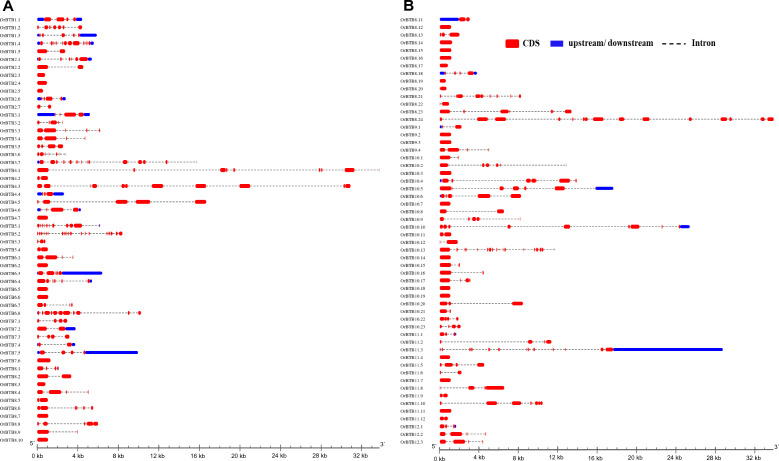
Graphical representation of exon–intron organization of *OrBTB* genes divided into two groups for clarity. (**A**) Graphics showing *OrBTB*1.1 to *OrBTB*8.10. (**B**) Graphics showing *OrBTB*8.11 to *OrBTB*12.3. Introns = horizontal red boxes; UTRs = boxes in blue; introns = dashed lines. Introns and exons were drawn in scale relative to their actual length. Figure was generated using GSDS web server version 2.0.

Members of the BTB/POZ gene family of *O. rufipogon* were widely diverse in terms of intron–exon architectures, with exon numbers ranging from 1 to 19 (Fig. 4). The majority of genes (70%, 77 out of 110 genes) had a relatively simple intron–exon architecture with one to four exons. Of these, 26 genes had a single exon. Complex intron–exon architectures (ten or more exons) were also evident across the gene family, although in a much smaller number of genes (10%; 11 out of 110). The most complex was evident in *OrBTB5*.2/ORUFI05G16760 with 19 exons.

Peptide motifs are highly conserved amino acid sequences with potential protein structural and functional implications. Analysis by MEME revealed ten significant peptide motifs among the BTB/POZ proteins, designated as motif-1 to motif-10 (Supplementary Fig. 2). Particularly noteworthy were motif-2 (ETFAAHRCVLAARSPVF) and motif-3 (IDDMEPAVFKALLHFIYTDSLP), occurring 84-times and 73-times, respectively. Motif-8 (YLRDDCFTIRCDVTVV) represented the least frequently occurring, only 57-times (Supplementary Fig. 3). Overall, trends in intron–exon architecture and conservation of peptide motifs suggest that apart from the important role of gene duplication, the evolution of the BTB/POZ protein family of *O. rufipogon* likely had significant contributions from domain-shuffling and exon-shuffling, which may have important implications to functional specialization.

### Synteny of BTB/POZ tandem arrays across flowering plants

Evolutionary dynamics of the BTB/POZ genes of *O. rufipogon* were investigated to understand the role of gene duplication and selection on gene family expansion. Trends revealed by the analysis of amino acid sequence homology showed 24 BTB/POZ gene pairs representing co-paralogs (Supplementary Table 3). Of these, eight genes appeared to have originated from segmental duplication, which gave rise to four gene pairs (Fig. 1B). These results indicate that the continuous expansion of this large gene family in *O. rufipogon* was facilitated primarily by tandem duplication and secondarily by non-tandem duplication. It is noteworthy that the largest number of co-paralogs are located in the chromosome-10 (18 genes) and chromosome-8 (10 genes) clusters. Analysis of non-synonymous/synonymous ratios of the duplicated gene pairs indicated that these duplicated BTB/POZ genes have undergone purifying selection based on the Ka/Ks < 1^76^. The average divergence time estimated for the duplicated genes was 54.2 MYA (Supplementary Table 3).

**Figure 5:**
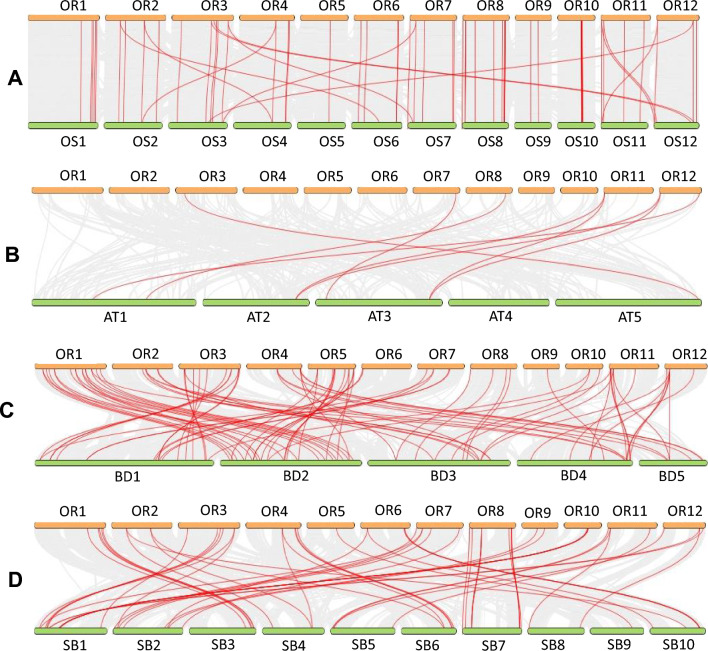
Analysis of collinearity between the *OrBTB* family with representative species of the monocot and dicot groups of plants. Yellow lines represent *Oryza rufipogon* (OR) chromosomes, while the green lines represent (**A**) *Oryza sativa* ssp. *japonica* (OS; IRGSP-1.0), (**B**) *Arabidopsis thaliana* (AT; TAIR10), (**C**) *Brachypodium distachyon* (BD; *Brachypodium_distachyon*_v3.0), and (**D**) *Sorghum bicolor* (SB; Sorghum_bicolor_NCBIv3) chromosomes. The grey lines in the background represent the collinear blocks in the genome of *Oryza rufipogon* and other species, while the red lines indicate the syntenic BTB gene pairs. Figure was generated using dual synteny plot option of Tbtool software.

Synteny reflects the magnitude by which large gene families evolved independently in different lineages. Trends revealed from dual synteny analysis with four other reference genomes representing a wide diversity across flowering plants such as *Oryza sativa* ssp. *japonica* (domesticated from *O. rufipogon;* IRGSP-1.0), *Arabidopsis thaliana* (dicot lineage; TAIR10), *Brachypodium distachyon* (small-genome monocot distantly related to *Oryza*; *Brachypodium_distachyon*_v3.0), and *Sorghum bicolor* (large-genome diploid monocot as distant lineage to *Oryza*; Sorghum_bicolor_NCBIv3) indicated that colinear gene clusters indeed evolved from common ancestral gene copies (Fig. 5). As expected, the largest number of syntenic *O. rufipogon* BTB/POZ gene clusters were observed in its domesticated version *Oryza sativa* ssp. *japonica* with a total of 71 syntenic loci. Continuous breakage of syntenic blocks was evident across the comparative evolutionary panel with a significant decline in synteny with increasing phylogenetic distance, i.e., 56 conserved syntenic loci in *B. distachyon* and *S. bicolor,* and only 8 in the dicot lineage represented by *A. thaliana* (Supplementary Table 4).

It is noteworthy that all eight syntenic loci between *O. rufipogon* and *A. thaliana* were also conserved with the two monocot lineages (*Sorghum*, *Brachypodium*). Members of these syntenic loci across the monocot and dicot lineages may provide an important gene set for developing Conserved Ortholog Set (COS) markers in comparative plant genomics studies, and particularly for the mining of novel BTB/POZ alleles from *O. rufipogon*. Most importantly, dual synteny analysis revealed that five BTB/POZ orthologs of *O. rufipogon* are conserved in *Brachypodium* (*OrBTB4.7/ORUFI04G29880*, *OrBTB8.8/ORUFI08G07520*, *OrBTB8.20/ORUFI08G22910, OrBTB10.3/ORUFI10G11060, OrBTB11.6/ORUFI11G21740*) and six in *Sorghum* (*OrBTB4.7/ORUFI04G29880, OrBTB6.6/ORUFI06G25940, OrBTB8.8/ORUFI08G07520, OrBTB8.22/ORUFI08G22930, OrBTB10.2/ORUFI10G11050, OrBTB11.4/ORUFI11G21310*), but not in its domesticated species. This loss of orthologs in *O. sativa* suggests putative gene copies that have been left behind in the wild gene pool during domestication.

### Upstream sequences of *O. rufipogon* BTB/POZ genes

**Figure 6:**
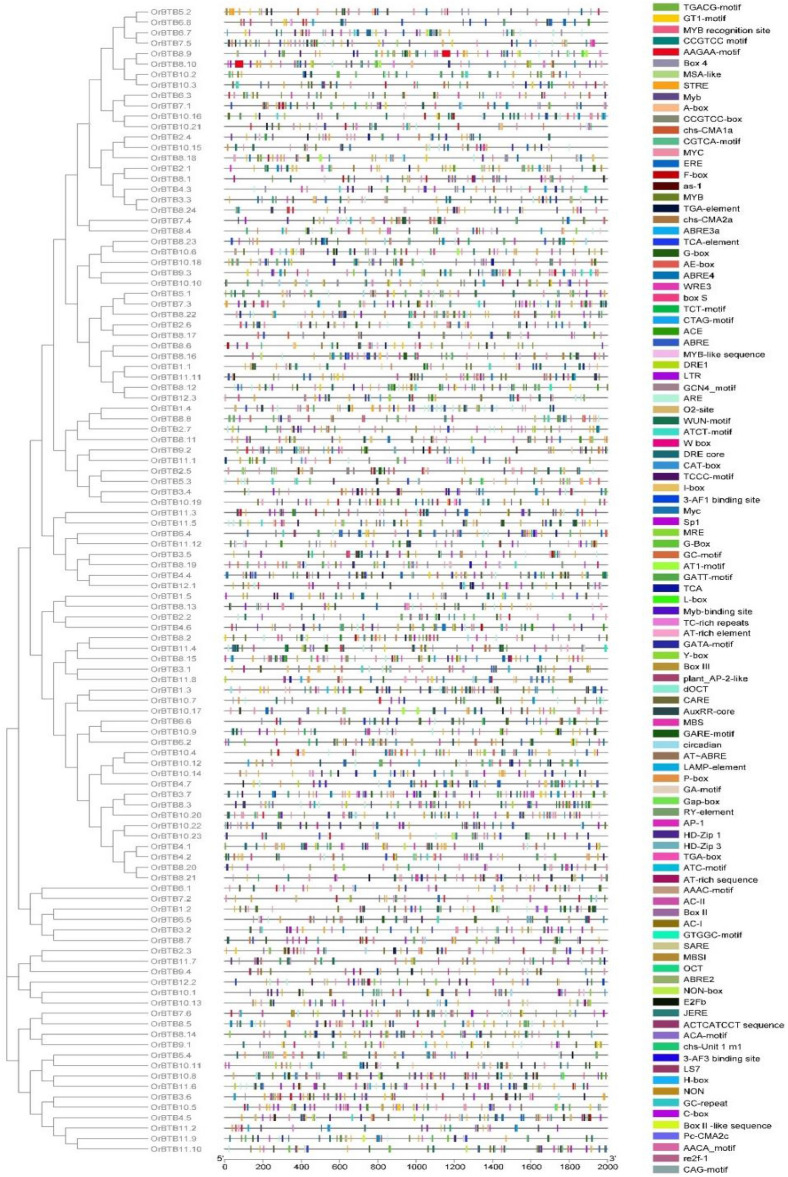
Distribution of significantly enriched sequence motifs representing putative cis-elements across the upstream regions of *OrBTB* genes. Potential biological implications of conserved sequence motifs were inferred based on homology with known cis-elements in the PlantCare databases. The scale at the bottom represents the relative position of the putative cis-acting elements across the upstream regions of *OrBTB* gene loci. Figure was generated using Bisequence visualiser option of TBtool software.

A total of 102 unique classes of sequence motifs were identified across the − 2000-bp region of the BTB/POZ genes (Fig. 6). On average, genes contained more than 50 classes of putative cis-elements. The potential functional diversity represented by these putative cis-elements (PlantCare database) suggests multiple pathways by which the BTB/POZ gene family members are regulated. Out of the 110 BTB/POZ family members in *O. rufipogon*, the *OrBTB4.4/ORUFI04G17810* had the largest number of hits (n = 93) for known cis-elements, while the *OrBTB11.7/ORUFI11G21760* had the lowest (n = 29) (Supplementary Table 5). The vast majority of putative cis-elements appeared to be relevant to abiotic stress response mechanisms and hormonal signalling. This was further reiterated by the enrichment of GO keywords such as abiotic stress, jasmonic acid, abscisic acid, wound response, development, and combinatorial gene network regulation. Putative cis-elements of MYB-type transcription factors were the most significantly enriched (i.e., 484 MYB, 104 MYB-binding sites, 93 MYB-like sequences, 73 MYB recognition sites), suggesting key functions of MYB transcription factors in the cellular and biological processes by which BTB/POZ genes are either directly or indirectly involved^77–79^.

A strong association of BTB/POZ genes with general stress response pathways was implied by the enrichment of different classes of transcription factors involved in stress-mediated regulation, including 387 STRE (stress response element), 379 ABRE (ABA-responsive elements), 83 ABRE4, 83 ABRE3a, and 89 DRE (dehydration-responsive element). This enrichment of stress-responsive cis-elements is consistent with the reported functions of transcription factors in dehydration and osmotic^80–84^, oxidative^85^, heat^86^, cold^87,88^, and sugar starvation^89^ stresses. These observations further support the potential contributions of BTB/POZ diversification to the inherent stress tolerance potential of the wild species *O. rufipogon*^9,10^.

Another class of significantly enriched cis-elements among the BTB/POZ genes appeared to be targets of MYC transcription factors (399), particularly those involved in jasmonic acid (JA) signaling and wound-inducible responses during herbivory, pathogen invasion, and physical wounding. This trend is particularly evident from the enrichment of WOUND RESPONSIVE ELEMENTS (WRE), which occurred 133 times across the BTB/POZ gene family^90,91^. The G-box, which is known to regulate senescence-induced gene expression, occurred in 440 upstream locations of BTB/POZ genes^92^.

### Novelty of *O. rufipogon* BTB/POZ genes based on exon–intron structure

**Figure 7:**
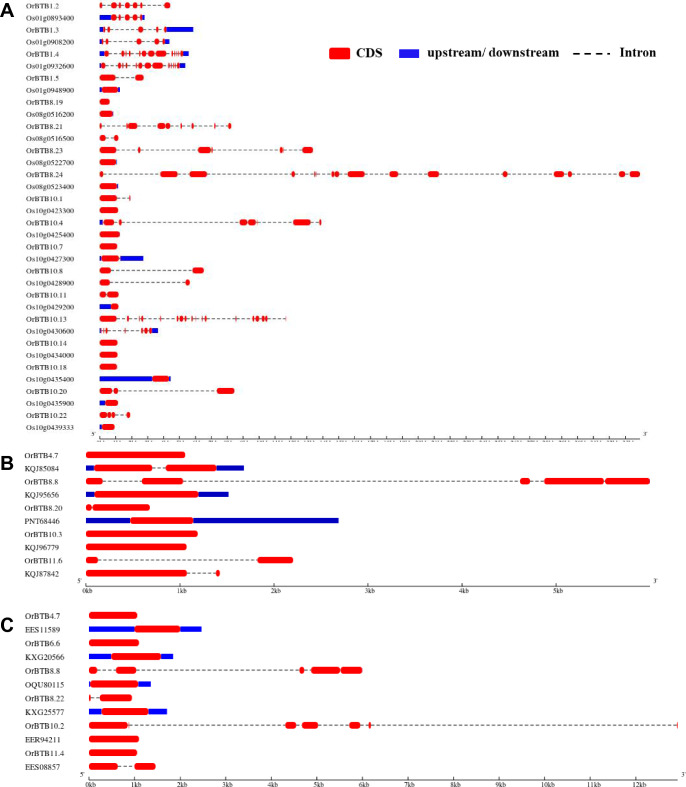
Comparison of structural differences within the coding regions of selected *OrBTB* and their orthologous genes. (**A**) *OrBTB* genes representing the COGs of chromosomes-01, -08, and -10 compared to their respective *O. sativa* ssp. *japonica* orthologs. (**B**) *OrBTB* orthologs that were lost in *O. sativa* ssp. *japonica* but remained conserved in *B. distachyon.* (**C**) *OrBTB* orthologs that were lost in *O. sativa* ssp. *japonica* but remained conserved in* S. bicolor.* Figures were generated using GSDS web server version 2 (http://www.gsds.gao-lab.org/).

We compared the intron–exon structures across orthologous *O. rufipogon* (*OrBTB*) and *O. sativa* ssp. *japonica* (*OsBTB*) genes that are located in three syntenic blocks located on chromosome-01 (four copies), chromosome-08 (four copies), and chromosome-10 (10 copies) in either one-to-one or one-to-few Clusters of Orthologous Groups (COGs) from available data in Ensembl Plants (https://plants.ensembl.org/index.html; Fig. 7A, Supplementary Tables 5, 6). In the COG from chromosome-01, we observed *OrBTB1.3/ORUFI01G44480* (5 exons) and *OrBTB1.4/ORUFI01G46370* (13 exons) as having the same exon–intron structures as their respective orthologous genes in *O. sativa* ssp. *japonica*, i.e., Os01t0908200 and Os01t0932600. In contrast, the respective orthologs of *OrBTB1.2/ORUFI01G43500* (7 exons) and *OrBTB1.5/ORUFI01G47340* (2 exons) in *O. sativa* ssp. *japonica*, i.e., Os01t0893400 (5 exons) and Os01t0948900 (1 exon) appeared to have diversified through the loss of exons in the domesticated genome.

Similarly, in chromosome-08 COG, the *O. sativa* ssp. *japonica* gene *Os08t0516200* had maintained the same intron–exon structure (1 exon) as its ortholog in *O. rufipogon, OrBTB8.19/ORUFI08G22900* (1 exon). However, the three other *O. sativa* genes in this cluster, i.e., *Os08t0516500* (2 exons), *Os08t0522700* (1 exon), *Os08t0523400* (1 exon), appeared to have drastic exon reduction relative to their respective *O. rufipogon* orthologs which maintained 9 exons (*OrBTB8.21/ORUFI08G22920*), 7 exons (*OrBTB8.23/ORUFI08G23440*), and 16 exons (*OrBTB8.24/ORUFI08G23450*).

We further observed a high degree of variability in intron–exon conservation in the chromosome-10 syntenic block between *O. rufipogon* and *O. sativa* ssp. *japonica*. Of the ten COGs in this block, only two orthologous pairs, i.e., *O. sativa*-*Os10t0428900* with *O. rufipogon*-*OrBTB10.8/ORUFI10G11150* (2 exons) and *O. sativa-Os10t0434000* with *O. rufipogon-OrBTB10.14/ORUFI10G11410* (1 exon) had conserved exon–intron structures. Two other COGs in this cluster, i.e., *OrBTB10.7/ORUFI10G11140* (1 exon) to *Os10t0427300* (2 exons), and *OrBTB10.18/ORUFI10G11500* (1 exon) to *Os10t0435400* (4 exons) appeared to have diversified through gain of exons in the domesticated genome. Six other COGs in this cluster showed indications of exon losses during domestication, i.e., *OrBTB10.1/ORUFI10G11040* (2 exons) to *Os10t0423300* (1 exon), *OrBTB10.4/ORUFI10G11080* (8 exons) to *Os10t0425400* (1 exon), *OrBTB10.11/ORUFI10G11190* (3 exons) to *Os10t0429200* (1 exon), *OrBTB10.13/ORUFI10G11230* (17 exons) to *Os10t0430600* (7 exons), *OrBTB10.20/ORUFI10G11550* (4 exons) to *Os10t0435900* (1 exon), and *OrBTB10.22/ORUFI10G11820* (4 exons) to *Os10t0439333* (1 exon).

We also compared the gene body of the orthologous set of *OrBTB* genes that were lost in *Oryza sativa* ssp.* japonica* but present in *Brachypodium distachyon* and *Sorghum bicolor* (Fig. 7B,C)*.* The general trend indicated that both high and low degrees of conservation in BTB/POZ gene structures existed after the divergence of these three monocot species. For example, the respective orthologs of *OrBTB10.3/ORUFI10G11060* (1 exon) and *OrBTB11.6/ORUFI11G21740* (2 exons) in *B. distachyon* (*KQJ96779, KQJ87842*) maintained a conserved exon–intron structure. Similarly, the respective orthologs of *OrBTB4.7/ORUFI04G29880, OrBTB6.6/ORUFI06G25940, OrBTB8.22/ORUFI08G22930* in *S. bicolor* (EES11589, KXG20566, KXG25577) all had maintained their conserved single exon structures after speciation. There were also examples of exon losses among BTB/POZ genes as a consequence of speciation of *O. rufipogon*, *B. distachyon*, and *S. bicolor*. For example, loss or gain of exons were evident between *OrBTB4.7/ORUFI04G29880* (1 exon), *OrBTB8.8/ORUFI08G07520* (5 exons), and *OrBTB8.20/ORUFI08G22910* (2 exons) and their respective orthologs in *S. bicolor* with 2 (KQJ85084), 1 (KQJ95656), and 1 (PNT68446) exons, respectively. The respective orthologs of *OrBTB8.8/ORUFI08G07520* (5 exons) and *OrBTB10.2/ORUFI10G11050* (7 exons) had a higher number of exons than their Sorghum counterparts OQU80115 and EER94211, each with only a single exon.

These observations indicated a generally higher similarity of the COGs of *O. rufipogon*, *B. distachyon*, and *S. bicolor* amongst each other in terms of exon–intron structure compared to COGs in the direct descendant of *O. rufipogon*–*O. sativa* ssp. *japonica*. These observations further suggest that differences between *O. rufipogon* (progenitor) and *O. sativa* ssp. *japonica* were due to domestication. Many BTB/POZ genes/alleles found in the wild have been conserved during monocot speciation but eliminated or altered by more intense selection during the domestication of *O. sativa* ssp. *japonica*. These results further support the hypothesis that the progenitor species of the modern-day cultivated rice is a potential source of novel alleles for allelic enrichment of the domesticated germplasm.

### Novelty of *O. rufipogon* BTB/POZ genes based on coding sequence variation

Orthologous BTB/POZ genes showed disparate dynamics in terms of mRNA and polypeptide length, with the rare exception of the perfectly homologous *O. rufipogon OrBTB10.14/ORUFI10G11410* and *O. sativa* ssp. *japonica* *Os10t0434000*, which encodes a highly conserved CDS of 1113-bp and polypeptide product of 370 amino acid residues (Supplementary Table 6). For the majority of orthologous gene pairs across the syntenic blocks between *O. rufipogon* and *O. sativa*, amino acid sequence alignment showed a variable percentage of identity (pid). For example, in the chromosome-10 cluster, *Os10t0435400* (49.6%) and *Os10t0425400* (64.2%) showed some of the lowest pid under coverage percentage (cov) of 91.6% and 38.8%, respectively, compared to their *O. rufipogon* ortholog *OrBTB10.18/ORUFI10G11500* and *OrBTB10.4/ORUFI10G11080* (Supplementary Fig. 4). In comparison with *Brachypodium* orthologs (5 orthologous loci), the pid ranged from 45.8 (*KQJ96779* versus *OrBTB10.3/ORUFI10G11060*, with a cov of 89.9) to 24.4 (*KQJ87842* versus *OrBTB11.6/ORUFI11G21740*, with a cov of 95.3). In comparison with *Sorghum* orthologs (6 orthologous loci), the pid ranged from 44.5 (*EES11589* versus *OrBTB4.7/ORUFI04G29880*, with a cov of 86.8 to 27.2 (*OQU80115* versus *OrBTB8.8/ORUFI08G07520* with a cov of 91.7) (Supplementary Fig. 5).

### Upstream regulatory signatures of orthologous BTB/POZ genes

**Figure 8:**
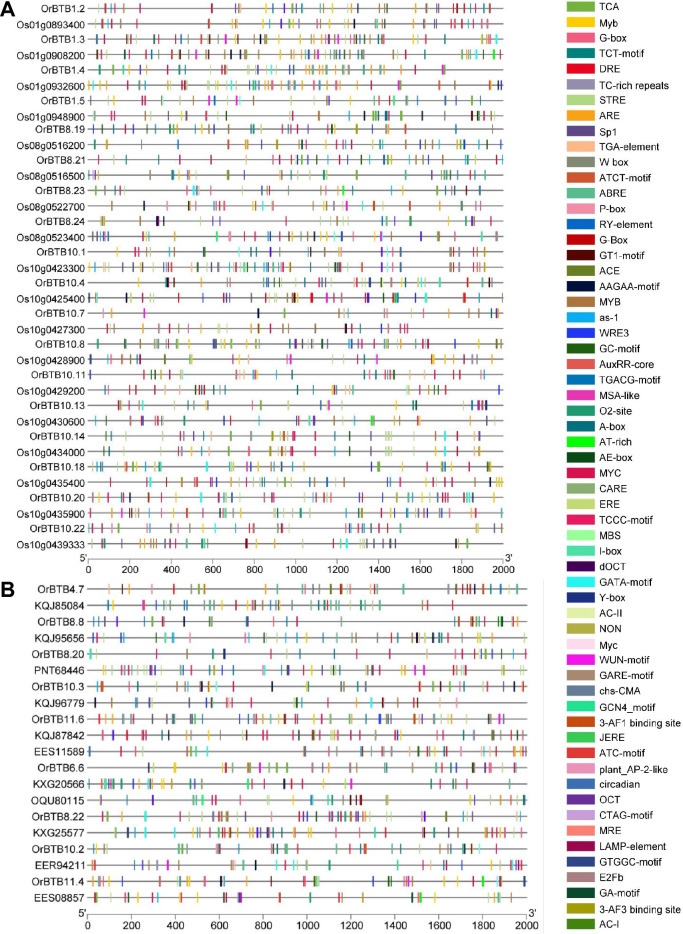
Diagram illustrating the loss or gain of putative cis-elements in *OrBTB* genes. (**A**) *OrBTB* orthologs on chromosomes-01, -08, and -10 compared to their *O. sativa* ssp. *japonica* orthologous. (**B**) *OrBTB* orthologs that were lost in *O. sativa* ssp. *japonica* but remained conserved in *B. distachyon* and *S. bicolor*. Figure was generated using Bisequence visualiser option of TBtool software.

The regulatory sequences of orthologous *O. rufipogon* and *O. sativa* ssp. *japonica* BTB/POZ genes also exhibited significant variation. Trends in the COGs of a given syntenic block showed differential patterns of enrichment for various types of cis-elements. For example, in chromosome-01 COGs, the *O. rufipogon* orthologs had a total of 176 putative cis-elements while the *O. sativa* ssp. *japonica* orthologs had 216 putative cis-elements (Fig. 8A, Supplementary Table 7). The ten most predominant classes of cis-elements among *O. sativa* ssp. *japonica* orthologs include the MYB (n = 35), ABRE (n = 24), G-box (n = 23), ARE (n = 15), STRE (n = 15), MYC (n = 13), TGACG-motif (n = 10), as-1 (n = 10), MBS (n = 6), and DRE (n = 5). In *O. rufipogon*, the ten most predominant cis-elements were the MYB (n = 26), ABRE (n = 19), STRE (n = 17), G-box (n = 17), ARE (n = 12), MYC (n = 8), TGACG-motif (n = 1), as-1 (n = 6), MBS (n = 5), and WUN (n = 5) (Fig. 8A).

Similarly, dissection of COGs in chromosome-08 cluster showed a total of 191 putative cis-elements in *O. sativa* ssp. *japonica* and 163 in *O. rufipogon*. The ten most predominant classes in *O. sativa* ssp. *japonica* orthologs are MYB (n = 37), ABRE (n = 25), G-box (n = 17), TGACG-motif (n = 16), as-1 (n = 16), MYC (n = 12), ARE (n = 7), MBS (n = 7), WRE3 (n = 6), and TCA (n = 6). Among *O. rufipogon* orthologs, the most predominant classes were MYB (n = 27), MYC (n = 17), as-1 (n = 13), TGACG (n = 13), WRE3 (n = 10), ABRE (n = 10), STRE (n = 8), G-box (n = 7), TCA (n = 5), and ARE (n = 6). COGs in chromosome-10 clusters showed generally similar trends of differential cis-element enrichment between the *O. sativa* ssp. *japonica* and *O. rufipogon* orthologs.

Inter-generic comparison (*O. rufipogon* vs. *S. bicolor* vs. *B. distachyon*) of upstream sequences of orthologous *OrBTB* genes lost in *Oryza sativa* ssp. *japonica* relative to *Brachypodium distachyon* and *Sorghum bicolor* revealed that *O. rufipogon* orthologs have higher enrichment of cis-elements (n = 389) than *S. bicolor* (n = 285) and *B. distachyon* (n = 322) (Fig. 8B, Supplementary Table 7). This suggested that based on intra-generic history (i.e., domestication = *O. rufipogon* vs. *O. sativa* ssp. *japonica*) and inter-generic history (i.e., speciation = *Oryza* vs. *Sorghum* vs. *Brachypodium*), the regulatory mechanisms operating on orthologous BTB/POZ genes have diverged considerably due to speciation and domestication. Similar to the trends revealed by gene sequence and structure comparison, novel alleles that appeared to have distinct regulatory mechanisms are the potential features of *O. rufipogon* BTB/POZ genes relative to *O. sativa* ssp. *japonica* orthologs.

### Potential role of miRNAs in the regulation of *O. rufipogon* BTB/POZ genes

Prediction of putative miRNAs with possible roles in regulating BTB/POZ genes suggested that 95 members of *O. rufipogon* BTB/POZ gene family are likely targets of 392 miRNAs belonging to more than 200 families (Supplementary Table 7). This initial set of candidate miRNAs was further reduced by false-positive filtration at a cut-off expectation score of 3.5, revealing 52 high-confidence candidate miRNA-target sites on BTB/POZ genes for 31 miRNA species (Supplementary Fig. 6). Of the 36 BTB/POZ genes that appeared to be regulated post-transcriptionally by miRNAs, four (*OrBTB4.5/ORUFI04G27660, OrBTB4.7/ORUFI04G29880, OrBTB8.23/ORUFI08G23440, OrBTB10.11/ORUFI10G11190*) had the highest number of potential miRNA target sites, with three sites each. Another set of eight BTB/POZ genes (*OrBTB1.3/ORUFI01G44480, OrBTB3.7/ORUFI03G38580, OrBTB4.1/ORUFI04G02660, OrBTB6.8/ORUFI06G26390, OrBTB7.6/ORUFI07G26650, OrBTB8.13/ORUFI08G07650, OrBTB8.2/ORUFI08G02030, OrBTB8.21/ORUFI08G22920*) had two putative target sites. The rest of the 24 BTB/POZ genes contained single putative miRNA-target sites.

Our analysis revealed that *miR5075* (*phytohormone synthesis*), *miR5809* (regulation of *finger transcription factors and subtilisin*), and *miR2927* (*plant growth*) appeared to be the most prominent post-transcriptional regulators of BTB/POZ genes with eight, six, and four targets, respectively. We also observed that all mRNA cleavage targets of miRNA5075 in *OrBTB1.1/ORUFI01G35500, OrBTB1.5/ORUFI01G47340, OrBTB3.2/ORUFI03G07690,* and *OrBTB6.8/ORUFI06G26390* clustered in the same phylogenetic group (Fig. 2), consistent with the highly conserved nature of miRNA target sites.

### Gene ontology and protein–protein interaction analysis

Gene Ontology (GO) enrichment across the 110 members of the BTB/POZ gene family of *O. rufipogon* indicated eight high-level GO terms for biological process, i.e., protein binding, histone acetyltransferase activity, peptide-lysine-*N*-acetyltransferase activity, peptide *N*-acetyltransferase activity, transcription coregulator activity, *N*-acetyltransferase activity, *N*-acyltransferase activity, and acetyltransferase activity (Supplementary Fig. 7). Enrichment trends for molecular function indicated that 13 genes were distinctly involved in protein ubiquitination, protein modification by small protein conjugation, and protein modification by small protein conjugation or removal. Enrichment trends for cellular components revealed three BTB/POZ genes (*OrBTB1.2/ORUFI01G43500, OrBTB2.6/ORUFI02G24050*, *OrBTB4.4/ORUFI04G17810*) involved in mechanisms of host–pathogen interaction (Supplementary Fig. 7, Supplementary Table 9).

**Figure 9:**
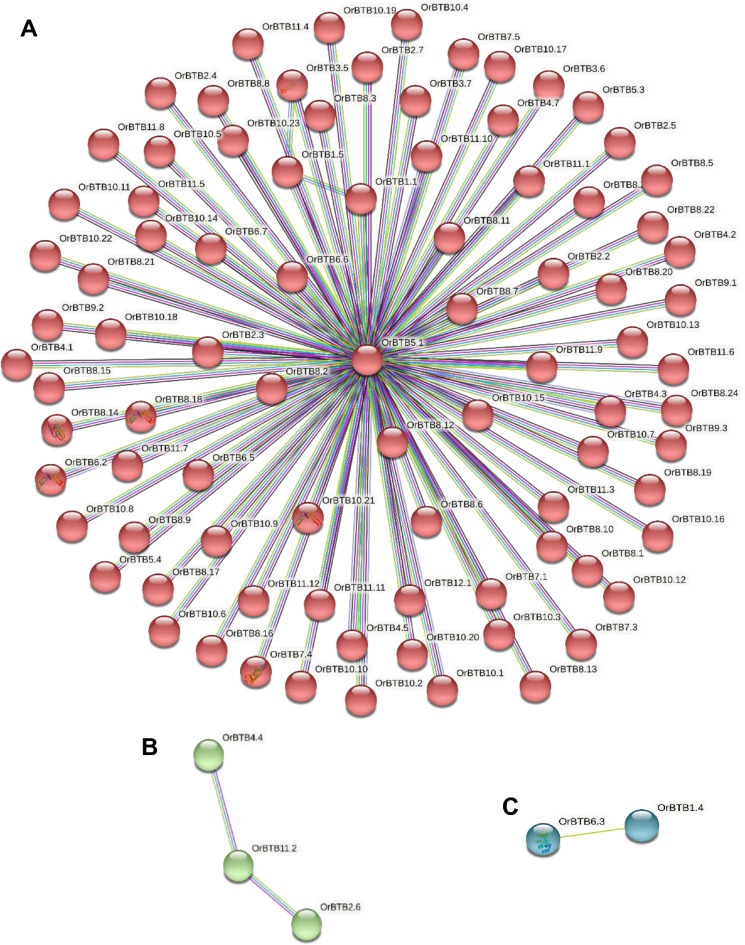
Map of predicted Protein–Protein Interaction (PPI) networks among *OrBTB* genes using Markov Cluster Algorithm (MCL). (**A**) Cluster-1 consists of 87 *OrBTB* network nodes. (**B**) Cluster-2 consists of three *OrBTB* nodes. (**C**) Cluster-3 consists of two *OrBTB* nodes. Figure was generated using STRING web server (https://www.string-db.org/).

Prediction and modeling of protein–protein interaction among the BTB/POZ protein family members of *O. rufipogon* using the STRING database and Markov Cluster Algorithm (MCL) revealed three major interaction clusters (Fig. 9). The largest (Cluster-1) consisted of 87 genes (nodes) with 88 interactions (edges) (Supplementary Table 10). The average local clustering coefficient in this cluster was 0.985*,* with *OrBTB 5.1/ORUFI05G13500* appearing to be the most likely central hub of the network. As the second-largest, Cluster-2 was comprised of three nodes (*OrBTB4.4/ORUFI04G17810, OrBTB11.2/ORUFI11G02560, OrBTB2.6/ORUFI02G24050*) and two edges, with an average local clustering coefficient of 0.667. The *OrBTB11.2/ORUFI11G02560* connected the other two nodes of this cluster. Cluster-3 was the smallest, having only two nodes with an average local clustering coefficient of 1.0 (Supplementary Table 10).

### Spatio-temporal expression of BTB/POZ genes

Publicly available transcriptome datasets of *O. rufipogon* included various tissue/organ (i.e., tiller bases, roots, leaf pulvini, leaf sheaths, nodes, culms, panicles > 5 cm) and environmental response (i.e., Fe deficiency, salt stress, cold stress) RNA-Seq libraries. Mining these datasets to profile the range of spatio-temporal expression across the BTB/POZ gene family showed substantial expression differences among the members.

**Figure 10:**
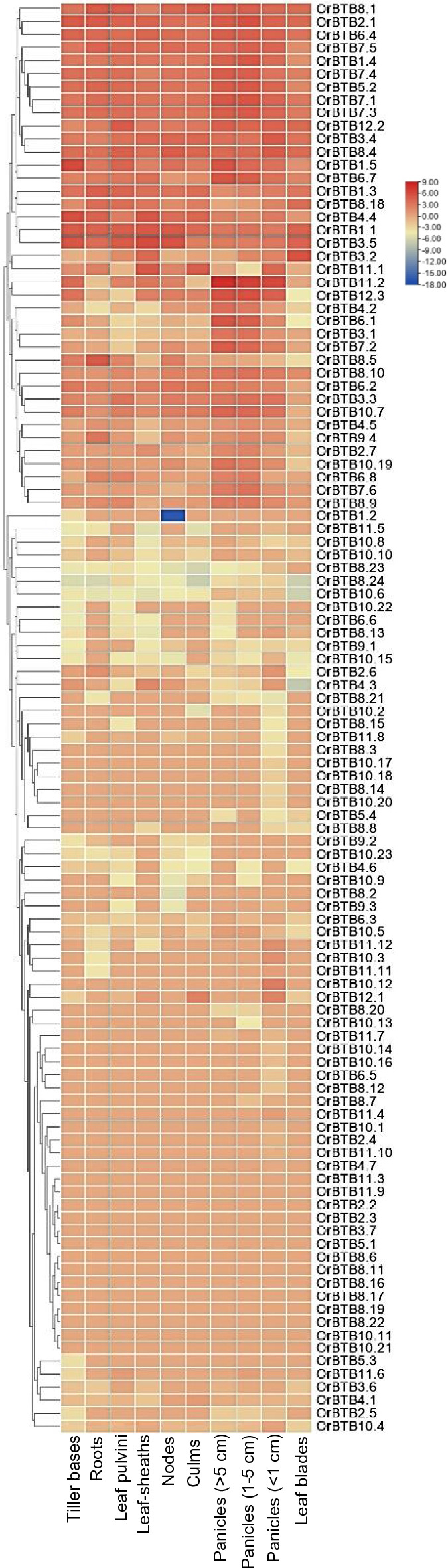
Spatial expression profiles of 110 *OrBTB* genes (i.e., tiller bases, roots, leaf pulvini, leaf sheaths, nodes, culms, panicles > 5 cm, panicles 1–5 cm, panicles < 1 cm, abd leaf blades based on the analysis of publicly available RNA-Seq datasets of *O. rufipogon* (*NCBI accession SRP151515*).

Hierarchical clustering of spatio-temporal expression showed that 40 of the 110 gene family members are expressed in all tissues/organs and 13 had non-detectable expression in all tissues/organs (Fig. 10). These observations implied varied functions of gene family members in cellular housekeeping processes as well tissue/organ-specific processes. For instance, 66 (tiller base), 57 (roots), 63 (leaf pulvini), 59 (leaf sheaths), 61 (nodes), 58 (culms), 62 (panicles, > 5 cm), 63 (panicles, 1–5 cm), 86 (panicles, < 1 cm), and 53 (leaf blades) members of the gene family had significantly higher tissue/organ-limited expression.

The majority of BTB/POZ genes exhibited specific patterns of upregulation or downregulation in certain tissues/organs, with the exception of *OrBTB1.2/ORUFI01G43500, OrBTB8.3/ORUFI08G02060, OrBTB8.24/ORUFI08G23450, OrBTB10.6/ORUFI10G11100, OrBTB4.3/ORUFI04G14290,* and *OrBTB10.2/ORUFI10G11050.* For instance, *OrBTB1.2* was downregulated in the nodes, suggesting its potential role in vegetative organ development and growth.

**Figure 11:**
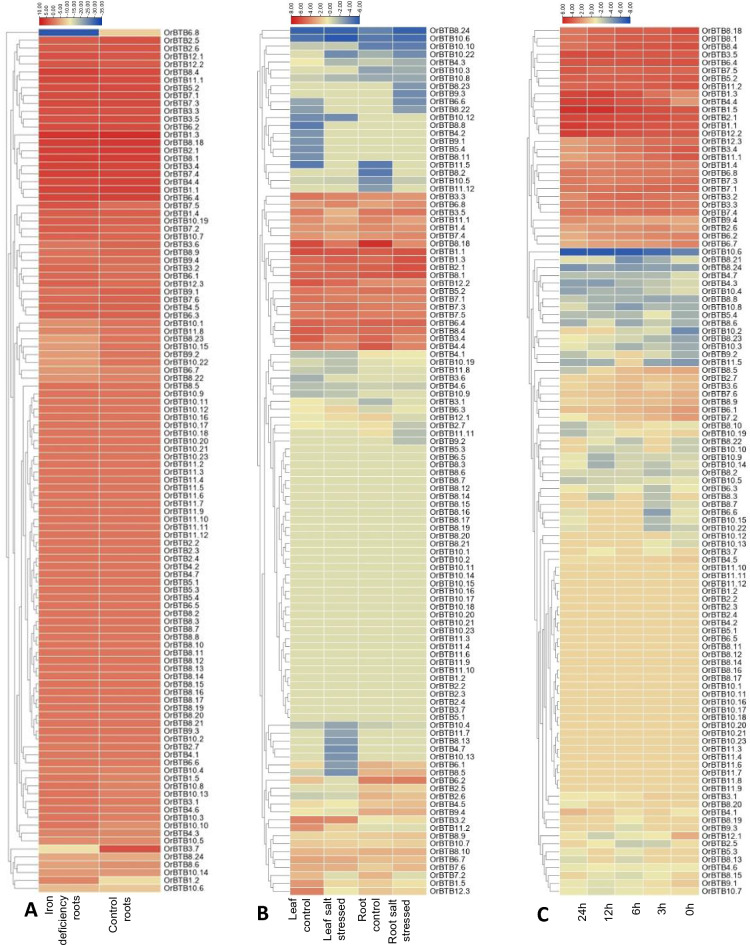
Effects of abiotic stresses on the expression of all 110 *OrBTB* genes based on the analysis of publicly available transcriptome datasets. (**A**) Expression patterns of *OrBTB* genes in the roots of *O. rufipogon* (EMBRAPA accession BRA 00004909-8) under iron deficiency (NCBI accession SRP198462). (**B**) Expression of *OrBTB* genes in leaves and roots of *O. rufipogon* seedlings under salinity stress at 200 mM NaCl (NCBI accession SRP063832 for salinity stress). (**C**) Expression of *OrBTB* genes in 3-day-old seedlings (accession Y12-4) under cold (4 °C) stress (NCBI *accession SRP251791*).

Certain BTB/POZ gene family members were clearly regulated in response to environmental stressors. For example, under Fe (iron) deficiency, 51 gene family members had constitutive expression while 60 others were differentially expressed in the roots (Fig. 11A). In particular, *OrBTB1.2/ORUFI01G43500* was upregulated by Fe deficiency while *OrBTB3.7/ORUFI03G38580* and *OrBTB 6.8/ORUFI06G26390* were downregulated. Salinity stress appeared to regulate the expression of 62 BTB/POZ genes in the leaves and 46 others in the roots (Fig. 11B). Notably, *OrBTB10.22/ORUFI10G11820, OrBTB8.13/ORUFI08G07650, OrBTB4.7/ORUFI04G29880,* and *OrBTB10.3/ORUFI10G11060* were downregulated in the leaves, while *OrBTB8.23/ORUFI08G23440, OrBTB9.3/ORUFI09G06920, OrBTB6.6/ORUFI06G25940* were downregulated in the roots. These observations indicated that the functional specification of different BTB/POZ gene family members as defined by the integration of various intrinsic and extrinsic signals, consistent with the trends revealed by cis-element and protein–protein interaction analyses.

BTB/POZ genes also appeared to be involved in low-temperature response. In particular, *OrBTB 1.3/ORUFI01G44480* and *OrBTB 4.4/ORUFI04G17810* showed continuous upregulation under cold stress (Fig. 11A).

**Figure 12:**
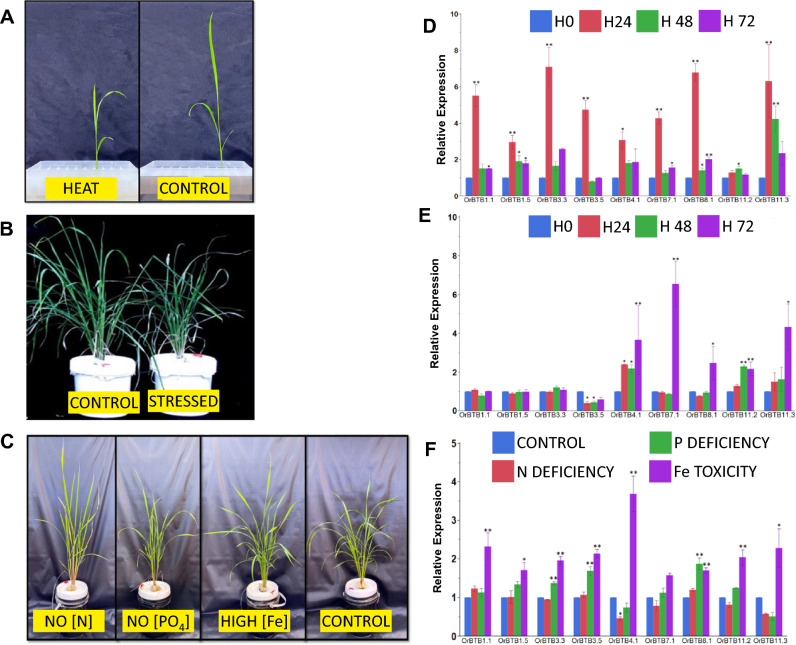
Phenotypic response of plants during (**A**) 7 days of heat stress, (**B**) 14 days of salt stress (**B**), and (**C**) 25 days of nutrient stress. Expression of *OrBTB* genes in response to (**D**) heat stress, (**E**) salinity stress, and (**F**) nutrient stress including nitrogen deficiency, phosphate deficiency, and iron toxicity. Gene expression analysis was performed by qRT-PCR and transcript levels are expressed as relative expression normalized relative to a constitutively expressed tubulin gene. Vertical bars indicate standard errors (n = 3).

Direct involvement of BTB/POZ genes in various stress response mechanisms provided the impetus for further validation of expression changes under different abiotic stress regimes by qRT-PCR (Fig. 12). The expression of nine representative genes, selected based on gene ontology, was profiled in *O. rufipogon* during exposure to nutrient and abiotic stress conditions. Results indicated that *OrBTB3.5/ORUFI03G29880* was consistently downregulated with progressive exposure to heat stress, while other gene family members (*OrBTB4.1/ORUFI04G02660, OrBTB7.1/ORUFI07G00140, OrBTB8.1/ORUFI08G00320, OrBTB11.2/ORUFI11G02560, OrBTB11.3/ORUFI11G12230*) were consistently upregulated after prolonged exposure to heat stress (Fig. 12).

Salinity stress rapidly but transiently induced several BTB/POZ genes, as evident from significant increases in transcript abundances as early as 24 h after the onset of stress. However, elevated transcript levels were not sustained during prolonged exposure suggesting that these genes were responsive to osmotic shock but were not likely adaptive in nature (Fig. 12). Additionally, a few other BTB/POZ genes, particularly *OrBTB11.3/ORUFI11G12230, OrBTB3.3/ORUFI03G26720, OrBTB3.5/ORUFI03G29880,* and *OrBTB8.1/ORUFI08G00320* had relatively late but stable upregulation, suggesting potential roles in adaptive mechanisms. Under nitrogen deficiency, *OrBTB3.3/ORUFI03G26720, OrBTB3.5/ORUFI03G29880, OrBTB8.1/ORUFI08G00320* and *OrBTB4.1/ORUFI04G02660* were upregulated*,* and *OrBTB3.3/ORUFI03G26720, OrBTB3.5/ORUFI03G29880, OrBTB8.1/ORUFI08G00320* were downregulated. Iron (Fe) toxicity stress was a potent inducer of BTB/POZ genes as all nine members of the expression cohort showed a significant upregulation (Fig. 12). This suggests that *O. rufipogon* has the potential for iron toxicity tolerance.

## Discussion

The importance of crop improvement for providing sufficient food and fiber to a growing world population is at the forefront of crop research. With local and global environmental fluctuations increasing and becoming more severe, the necessity for improving crop productivity in the face of these challenges becomes imperative. Towards this end, it is increasingly apparent that sourcing the genetic potentials of crop wild relatives (CWR) will be instrumental in improving agronomic traits^93^. As components of many germplasm collections worldwide, CWR provide a rich source of genic and allelic diversity for various traits important for enhanced stress tolerance. As such, the use of wide introgression to introduce beneficial exotic genes or alleles into the narrow gene pool of modern crop cultivars is promising^94^. Indeed, a recent pan-genome analysis of 66 accessions representing the *O. sativa–O. rufipogon* species complex revealed approximately 23 million sequence variants across the genome^95^.

Improvement of complex traits for enhanced fitness to adverse conditions requires the creation of novel genetic networks. It is in this molecular design that novel genes and/or alleles from CWR could prove useful for creating alternative regulatory pathways that are responsive to developmental and environmental signals. Spatio-temporal and developmental regulation of gene expression employs a mosaic of molecular mechanisms in which a myriad of individual gene components are assembled to create intricate synergies. Within this molecular paradigm of novel genes or alleles from CWR, we investigated the molecular and genomic structure of the BTB/POZ gene family in the wild progenitor of rice, *Oryza rufipogon.* Our analyses substantiate the potential usefulness of BTB/POZ genes from this CWR in creating novel regulatory networks for enhanced biological functions.

In plants, BTB/POZ genes are known for their roles in plant growth, development, and stress response. While the gene family has been characterized in model plants and crop species, the number of member varies across species. For instance, 149 in *O. sativa* ssp. *japonica*^46^, 80 in *Arabidopsis thaliana*^46^, 38 in *Solanum lycopersicum*^28^, 34 in *Solanum pennellii*^28^, and 49 in *Beta vulgaris*^48^*.* In this study, we identified 110 genes encoding BTB/POZ proteins in *Oryza rufipogon* genome. The differences in the number of BTB/POZ genes across species appeared to be due to lineage-specific expansion and contraction^39,96^.

### Structural characteristics of OrBTB genes

At the protein level, differences were represented in the placement and architecture of the domain and conserved motifs, all of which implied potential significance in functional specialization. Aside from the BTB/POZ domain, the *OrBTB* proteins had a number of additional domains, including the MATH, TAZ, Arm, Ank, Methyltransferase, BACK, NPH3, NPR1, PA, Peptidase, and F5/8 type C domains. The presence of multiple functional domains in *Oryza rufipogon* BTB/POZ proteins suggests that different subfamilies of the larger BTB/POZ protein family may provide wide functional capacity within the genic landscape of the *O. rufipogon* genome. *Oryza rufipogon*, like japonica rice and Arabidopsis, lacks the BTB-BACK-kelch and BTB zinc finger combinations, which make up a large portion of vertebrate BTB/POZ collections^39,46^.

### OrBTB paralogs and orthologs

We investigated gene duplications for the BTB/POZ proteins in the *Oryza rufipogon* genome and captured paralogs, including twenty-four co-paralogs having eight segmentally duplicated genes. These co-paralogs have gone through purifying selection during evolution, confirming that relaxed negative selection is a common characteristic of lineage-specific genes^97^. These genes, under neutral evolution, could serve as a repository for future genetic innovations^96^. To determine how well conserved the BTB/POZ genes were across related species, we revealed the extent of *OrBTB* synteny across monocots (*O. sativa* ssp. *japonica*, *Sorghum*, *Brachypodium*) and dicots (Arabidopsis). As expected, japonica rice contained the maximum number of syntenic genes with *O. rufipogon*, whereas *Sorghum* and *Brachypodium* had the same number, and Arabidopsis had the least number of syntenic genes. This observation indicated that BTB/POZ genes might have experienced species-specific duplications after the monocot-dicot divergence event. We identified nine novel BTB/POZ genes in the *Oryza rufipogon* genome having orthologs in *Sorghum* and *Brachypodium* but not in cultivated *O. sativa* ssp. *japonica*, revealing AA-genome lineage-specific expansion and contraction. Furthermore, intense purifying selective pressures appeared to have played a crucial factor in optimizing the adaptive capability of *Oryza* species to specific ecological niches during evolution.

### Function and regulation of BTB/POZ genes

The modular architectures of upstream regulatory sequences of genes are important windows on how gene regulation is interfaced with hormonal signaling in response to intrinsic and extrinsic signals for growth, development, reproduction, and adaptability^82^. In the upstream regulatory sequences of *OrBTB* genes, we revealed a range of frequently occurring cis-elements associated with hormonal and environmental signaling pathways, including the MYB, MYC, WRE, STRE, ABRE, DRE, and G-box elements. These findings suggest that *OrBTB* genes are involved in various signaling pathways relevant to stress-response mechanisms. Surveying for miRNA target sites can also provide another layer of crucial information for gene expression^98,99^. The specific pairing of miRNAs with *OrBTB* genes could change their expression dynamics through post-transcriptional gene silencing (PTGS). In this study, we found 31 miRNAs with 52 target sites on 36 *OrBTB* genes. Of these, *miR5075* targets several genes in the GRAS (GIBBERELLIN-ACID INSENSITIVE (GAI), REPRESSOR of GA1 (RGA), and SCARECROW SCR), ERF (Ethylene Responsive Factor), C2H2 (cysteine and histidine residues) transcription factor families^100^. Similarly, *miR5809* targets putative finger transcription factors and subtilisin^101^, while *miR2927* targets MORE AXILLARY GROWTH1 (MAX1) genes encoding cytochrome P450 monooxygenases^102,103^. Regarding abiotic stress responses, *miR414* targets DEAD-box helicases involved in salinity stress response^104^ and another rice gene (LOC_Os05g51830) that regulates flowering and heading delay^105^. These miRNA target sites in *OrBTB* genes suggest potential epigenetic control of certain *OrBTB* genes during plant development under stress.

### Novel OrBTB genes

Comparison of the selective *O. rufipogon OrBTB* genes with their orthologs in *O. sativa* ssp. *japonica*, *Brachypodium,* and *Sorghum* divulged substantially diverged basal cis-elements. The apparent exon losses and gains in the BTB/POZ orthologous genes suggest that these two processes may be key factors in speciation and domestication. As a paleopolyploid genome, several gene families are extended in *Oryza*, with duplicated genes diversified via neofunctionalization or subfunctionalization^106^. Amino acid sequence alignment of selected COGs and novel orthologs revealed nucleotide substitutions and indels in the *OrBTB/POZ* genes. Such divergence may have important role in adapting different plant species to diverse environments through altered gene regulation. We found one perfect homology of *OrBTB* genes in *O. sativa* ssp. *japonica* (*Os10t043400* orthologous to *OrBTB10.14*/ORUFI10G11410). This could be important for finer-scale spatiotemporal variation of genes that execute similar biological activities across the two species.

### Functional implications of the diversity in the OrBTB protein family

BTB/POZ proteins are one of the most prominent families of scaffold proteins known for their interactions with other signalling molecules^26^. Given that *OrBTB* proteins contained secondary domains along with the signature BTB/POZ domain, we attempted to establish a system-wide understanding of *OrBTB* protein functions by gene ontology (GO) enrichment and protein–protein interaction prediction. GO enrichment revealed that *O. rufipogon OrBTB* proteins have a multitude of potential cellular functions. The conserved BTB/POZ domain has been reported to interact with cullin3 (CUL3) to form functional E3 ligases, which mediate the ubiquitination and subsequent proteasomal degradation of target proteins^29,37,46,107^. In addition to the primary function of protein binding, they also act in host–pathogen interaction, chromatin remodelling, protein degradation, and catalyzing the transfer of acyl groups. Protein interaction network prediction suggests the association of the majority of *OrBTB* proteins into a single interactive cluster, implying that they could form molecular networks or possibly exhibit heterodimerization to create specific functions.

To further explore the role of the *OrBTB* protein family in plant growth, development, and stress responses, we mined the global expression pattern of *OrBTB* genes from publicly available transcriptomic datasets. Across tissues and stress conditions (iron deficiency, cold, salt), the *OrBTB* genes demonstrated differential expression, providing evidence of their involvement in different response mechanisms. Of interest, we found *OrBTB1.2* to be highly downregulated in the nodes of *O. rufipogon,* validating the reported function of certain *OrBTB* genes in regulating plant architecture. In domesticated maize, the *tru1* (tassels replace upper ears1), a BTB/POZ-encoding gene, is directly targeted by TB1 (TEOSINTE BRANCHED1) transcription factor to inhibit axillary branch formation. The *tru1* mutants have overcrowding and lodging phenotype^51^. The observed downregulation of *OrBTB1.2 *(*ORUFI01G43500*) suggests potential role in the overcrowded branched plant architecture, a salient characteristic of *O. rufipogon*.

We investigated the potential involvement of *OrBTB* genes in stress responses by examining the expression of selected gene family members under nutrient stress (i.e., nitrogen deficiency, phosphate deficiency, and iron toxicity), salinity, and heat. Results indeed showed significant changes in expression patterns between control and stressed plants across stress regimes. Under salinity stress, results showed that *OrBTB* genes were upregulated during the early stages of stress. A previous report showed that upregulation of *OsBTBZ1* (Os01g66890), a BTB domain-containing gene, confers salt tolerance in rice^108^. In *Arabidopsis thaliana*, overexpression of BTB protein 1 (*AtSIBP1;* At1g55760) has been shown to cause tolerance to salt stress through mechanisms that alter reactive oxygen species (ROS) dynamics^56^. Under heat stress, we found only one *OrBTB* gene to be downregulated, while five others were upregulated. It has been shown that BTB/POZ and MATH DOMAIN proteins (BPMs) are involved in the proteolysis of *DREB2A* transcription factors to modulate thermotolerance in Arabidopsis^57^. BTB/POZ genes have important roles in nitrogen use efficiency in plants^53,109^. Under nitrogen-deficient conditions, we observed that *OrBTB4.1* (*ORUFI04G02660*) was downregulated (Fig. 12F). BTB/POZ genes BT1 and BT2 in Arabidopsis and their orthologs in rice (*Os01g68020*) act as negative regulators of nitrate uptake via Calcium-dependent protein kinases, dependent on the calmodulin-binding domain at the C terminus. Overexpression of BT2 has been shown to reduce nitrogen use efficiency under low nitrate conditions^53^.

Our qRT-PCR data showed significant upregulation of several *OrBTB* genes during exposure to toxic concentration of iron. In apple, BTB-TAZ proteins (*MdBT1* and *MdBT2*) are known to regulate iron homeostasis through the degradation of basic helix-loop-helix (bHLH) transcription factors by forming a Cullin-RING ubiquitin ligase 3 complex^54^. We also observed significant upregulation of several *OrBTB* genes under phosphate deficiency. In Arabidopsis, the ETHYLENE OVERPRODUCER 1 (ETO1), a BTB/POZ protein, negatively regulate ethylene synthesis through the degradation of type-2 ACS (1-aminocyclopropane-1-carboxylic acid synthases) under phosphate deficiency^110–112^.

## Conclusion

To our knowledge, this study represents the first systematic dissection of BTB/POZ protein-encoding genes in *O. rufipogon.* Phylogenetic studies of the 110 *OrBTB* genes along with gene and protein architecture analysis, supported the high-degree of diversity across the gene family, suggesting genic and allelic novelty. Evidence of segmental duplication, purifying selection, and lineage-specific gene loss, critical for the evolution of *OrBTB* genes were supported by the results. Enrichment of various cis-regulatory elements and miRNA binding sites revealed that the expression of *OrBTB* genes is tightly regulated at the transcription and post-transcription levels. The distinct spatio-temporal expression of novel BTB/POZ orthologs could be highlighted by the basal developmental programming of diverging cis-regulatory information, intron–exon structure, and amino acid sequences. Protein–protein interaction prediction showed that OrBTB proteins collaborate to achieve specific cellular functions as part of a complex molecular regulatory landscape that controls development and stress responses in *O. rufipogon*. Analysis of Gene Ontology (GO) enrichment revealed diverse cellular, molecular and biological functions of *OrBTB* genes. The extent of diversity across the *O. rufipogon OrBTB/POZ* gene family is relatively large compared to that of the domesticated counterpart *O. sativa* ssp. *japonica*, suggesting that *O. rufipogon* is a potential source of allelic variation for a major class of regulatory genes with important agronomic functions.

### Supplementary Information

Supplementary Figure 1.

Supplementary Figure 2.

Supplementary Figure 3.

Supplementary Figure 4.

Supplementary Figure 5.

Supplementary Figure 6.

Supplementary Figure 7.

Supplementary Table 1.

Supplementary Table 2.

Supplementary Table 3.

Supplementary Table 4.

Supplementary Table 5.

Supplementary Table 6.

Supplementary Table 7.

Supplementary Table 8.

Supplementary Table 9.

Supplementary Table 10.

## Data Availability

The datasets analyzed during the current study are from the publicly available genome sequence of *O. rufipogon* at Ensembl Databases (https://plants.ensembl.org/Oryza_rufipogon/Info/Index) and Short Read Archive of the National Center for Biotechnology Information (https://www.ncbi.nlm.nih.gov/sra) accessions SRP151515, SRP198462, SRP063832, and SRP251791. All other data generated or analyzed during this study are included in this published article and its supplementary information files.
