# Supplementary material for: Novel genes and alleles of the BTB/POZ protein family in Oryza rufipogon
Source: Sci Rep. 2023 Sep 19;13:15466. doi: 10.1038/s41598-023-41269-0 (PMC10509276; doi:10.1038/s41598-023-41269-0)
Supplement: Supplementary file 1 — Supplementary Figure 1. [file 41598_2023_41269_MOESM1_ESM.pptx]

## Slide 1
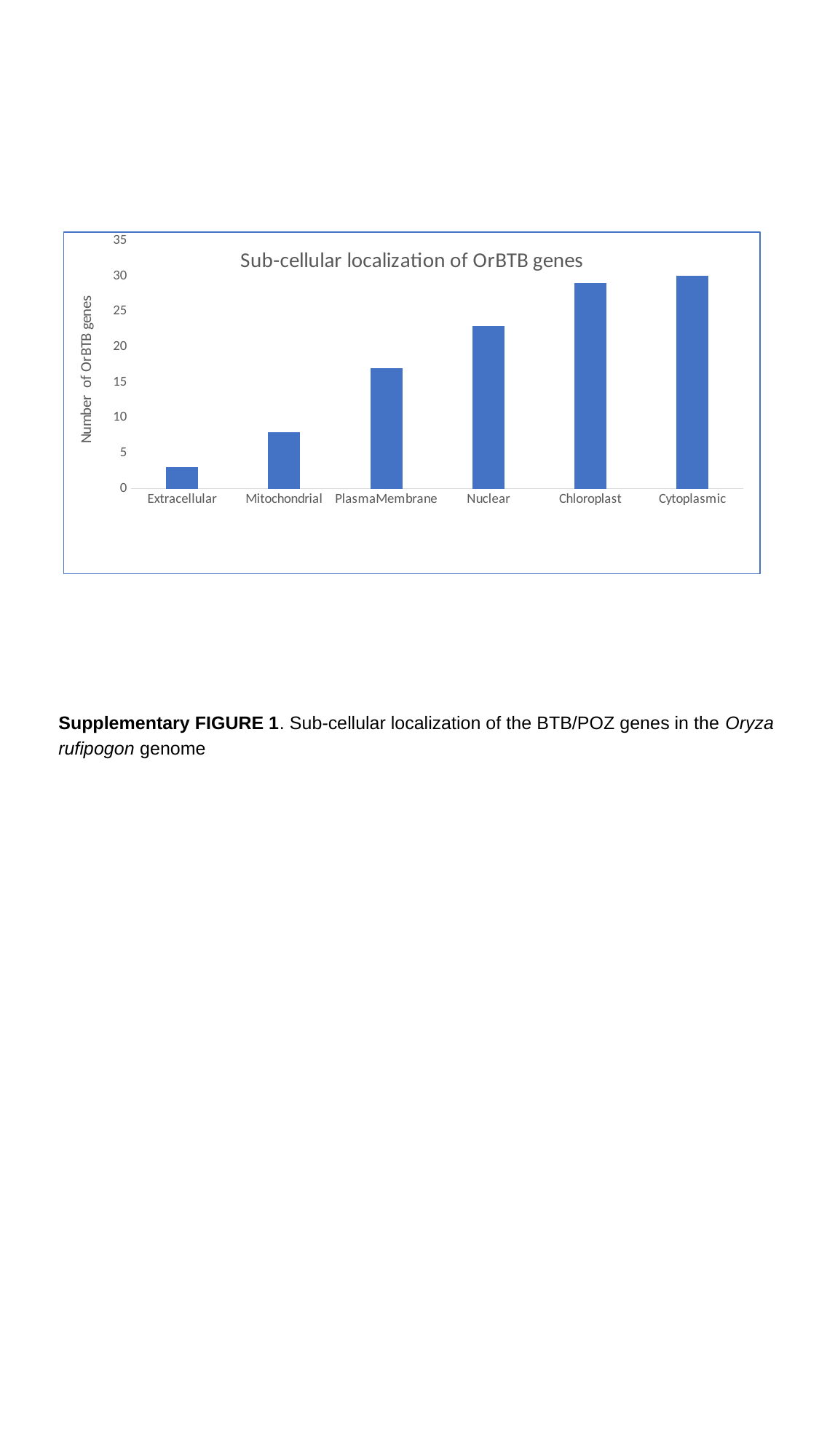

### Chart: Sub-cellular localization of OrBTB genes
| Category | |
|---|---|
| Extracellular | 3.0 |
| Mitochondrial | 8.0 |
| PlasmaMembrane | 17.0 |
| Nuclear | 23.0 |
| Chloroplast | 29.0 |
| Cytoplasmic | 30.0 |Supplementary FIGURE 1. Sub-cellular localization of the BTB/POZ genes in the Oryza rufipogon genome
