# Supplementary material for: Novel genes and alleles of the BTB/POZ protein family in Oryza rufipogon
Source: Sci Rep. 2023 Sep 19;13:15466. doi: 10.1038/s41598-023-41269-0 (PMC10509276; doi:10.1038/s41598-023-41269-0)
Supplement: Supplementary file 2 — Supplementary Figure 2. [file 41598_2023_41269_MOESM2_ESM.pptx]

## Slide 1
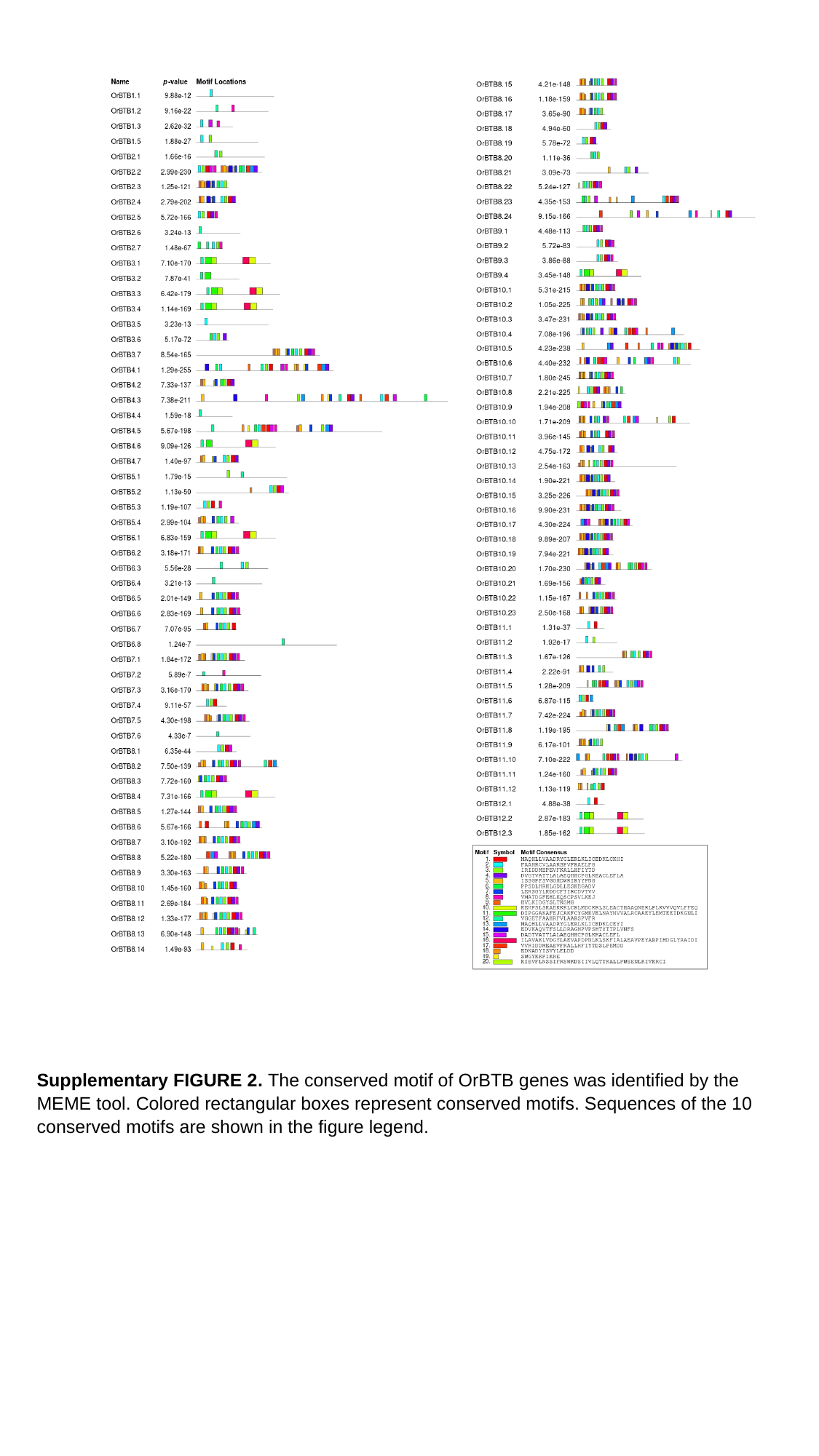

Supplementary FIGURE 2. The conserved motif of OrBTB genes was identified by the MEME tool. Colored rectangular boxes represent conserved motifs. Sequences of the 10 conserved motifs are shown in the figure legend.
