# Supplementary material for: Novel genes and alleles of the BTB/POZ protein family in Oryza rufipogon
Source: Sci Rep. 2023 Sep 19;13:15466. doi: 10.1038/s41598-023-41269-0 (PMC10509276; doi:10.1038/s41598-023-41269-0)
Supplement: Supplementary file 3 — Supplementary Figure 3. [file 41598_2023_41269_MOESM3_ESM.pptx]

## Slide 1
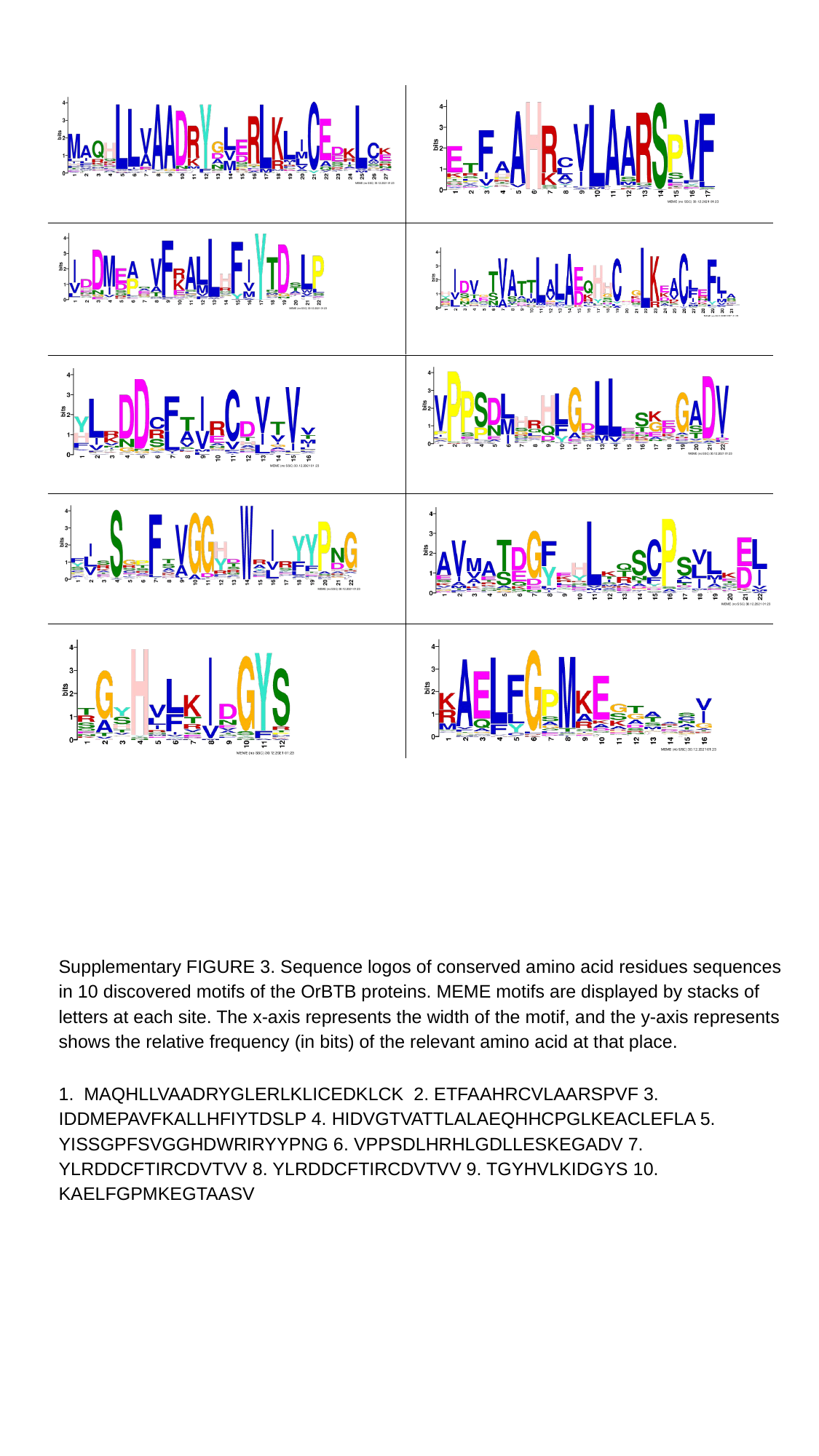

Supplementary FIGURE 3. Sequence logos of conserved amino acid residues sequences in 10 discovered motifs of the OrBTB proteins. MEME motifs are displayed by stacks of letters at each site. The x-axis represents the width of the motif, and the y-axis represents shows the relative frequency (in bits) of the relevant amino acid at that place.
1. MAQHLLVAADRYGLERLKLICEDKLCK 2. ETFAAHRCVLAARSPVF 3. IDDMEPAVFKALLHFIYTDSLP 4. HIDVGTVATTLALAEQHHCPGLKEACLEFLA 5. YISSGPFSVGGHDWRIRYYPNG 6. VPPSDLHRHLGDLLESKEGADV 7. YLRDDCFTIRCDVTVV 8. YLRDDCFTIRCDVTVV 9. TGYHVLKIDGYS 10. KAELFGPMKEGTAASV
