# Supplementary material for: Novel genes and alleles of the BTB/POZ protein family in Oryza rufipogon
Source: Sci Rep. 2023 Sep 19;13:15466. doi: 10.1038/s41598-023-41269-0 (PMC10509276; doi:10.1038/s41598-023-41269-0)
Supplement: Supplementary file 5 — Supplementary Figure 5. [file 41598_2023_41269_MOESM5_ESM.docx]

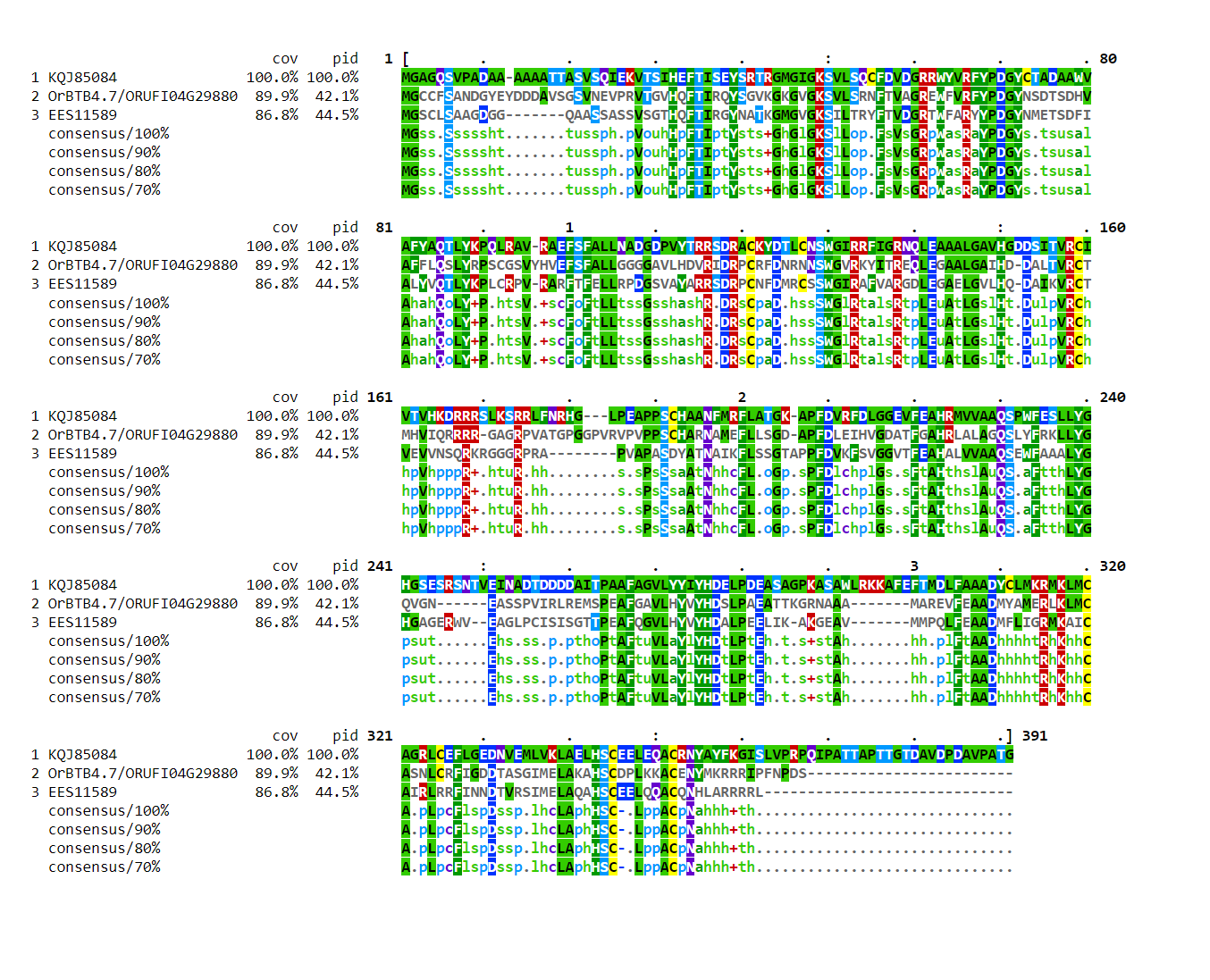


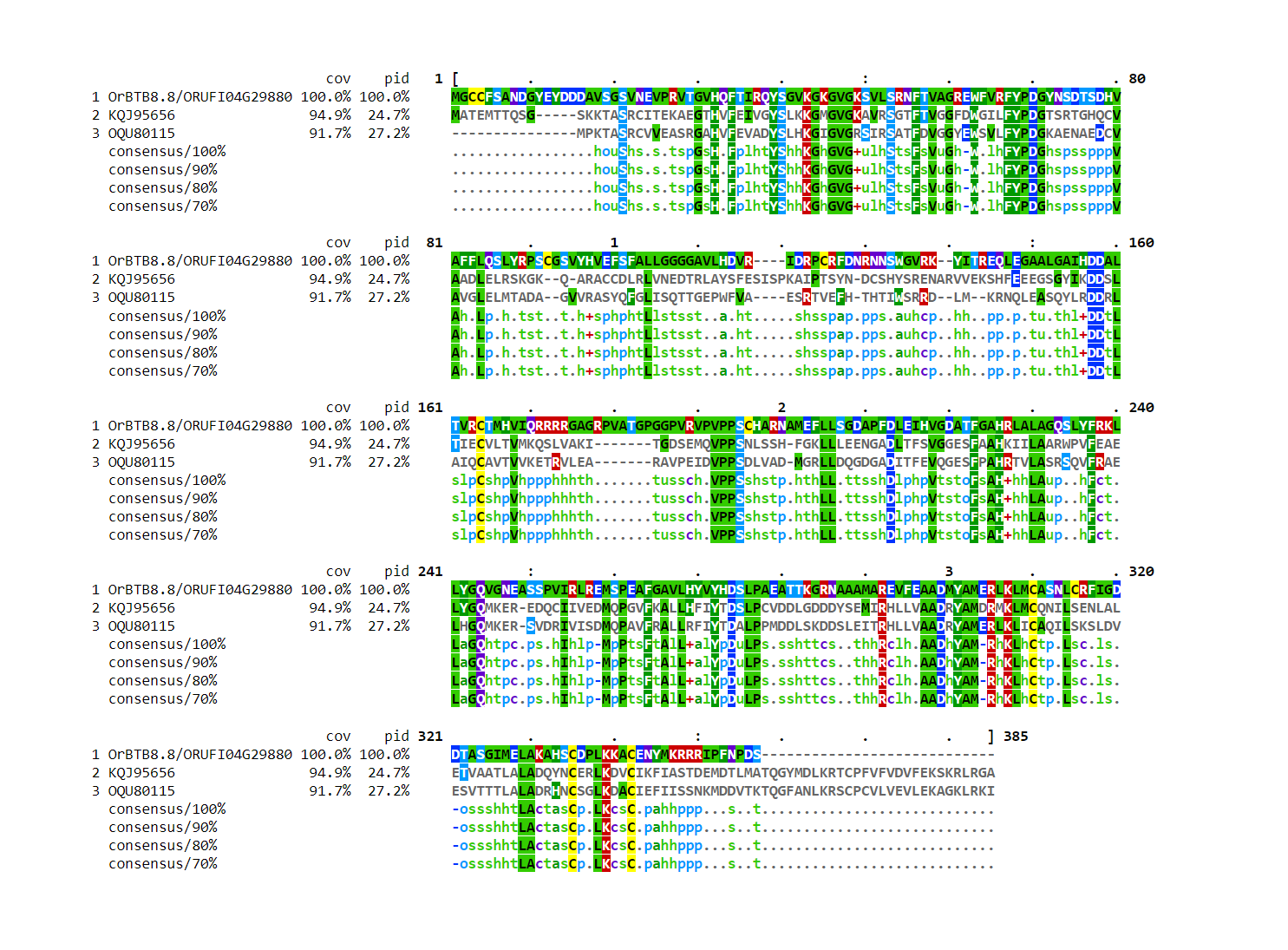


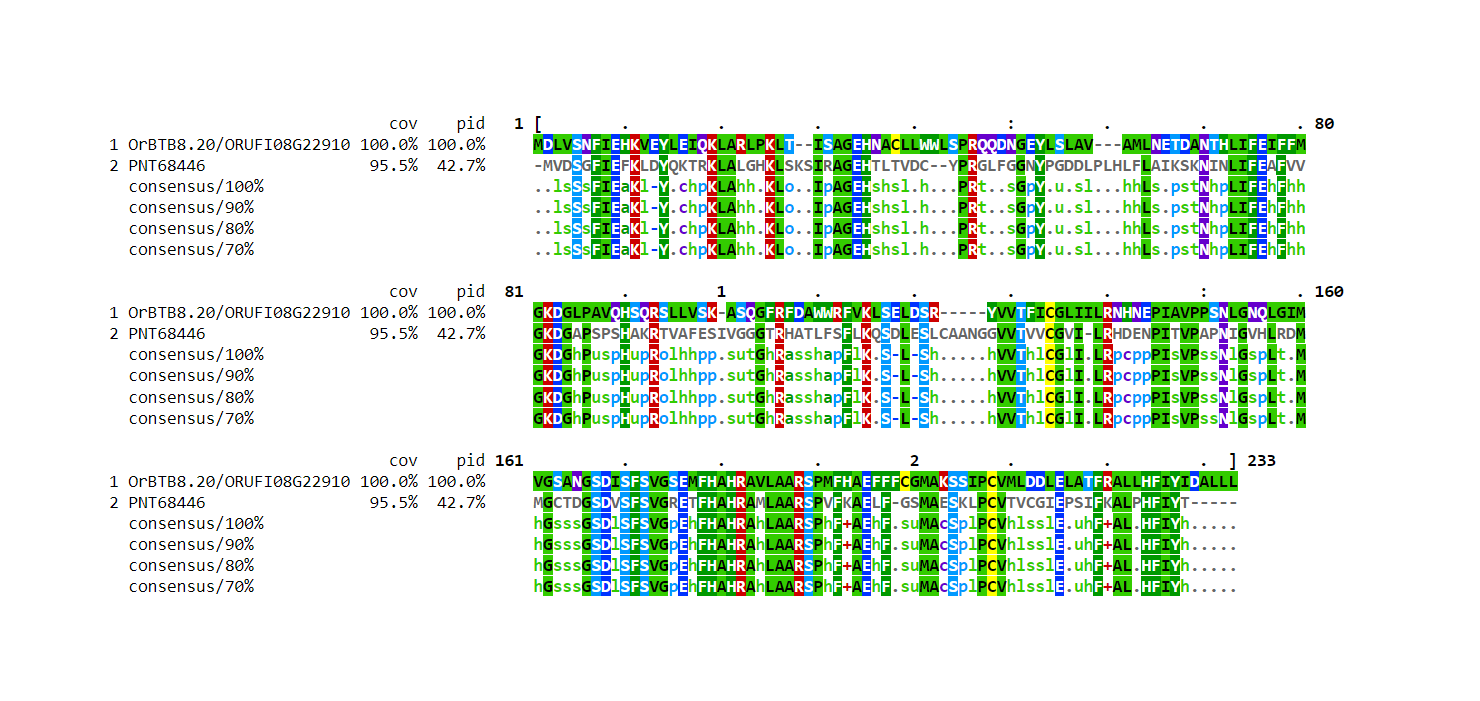


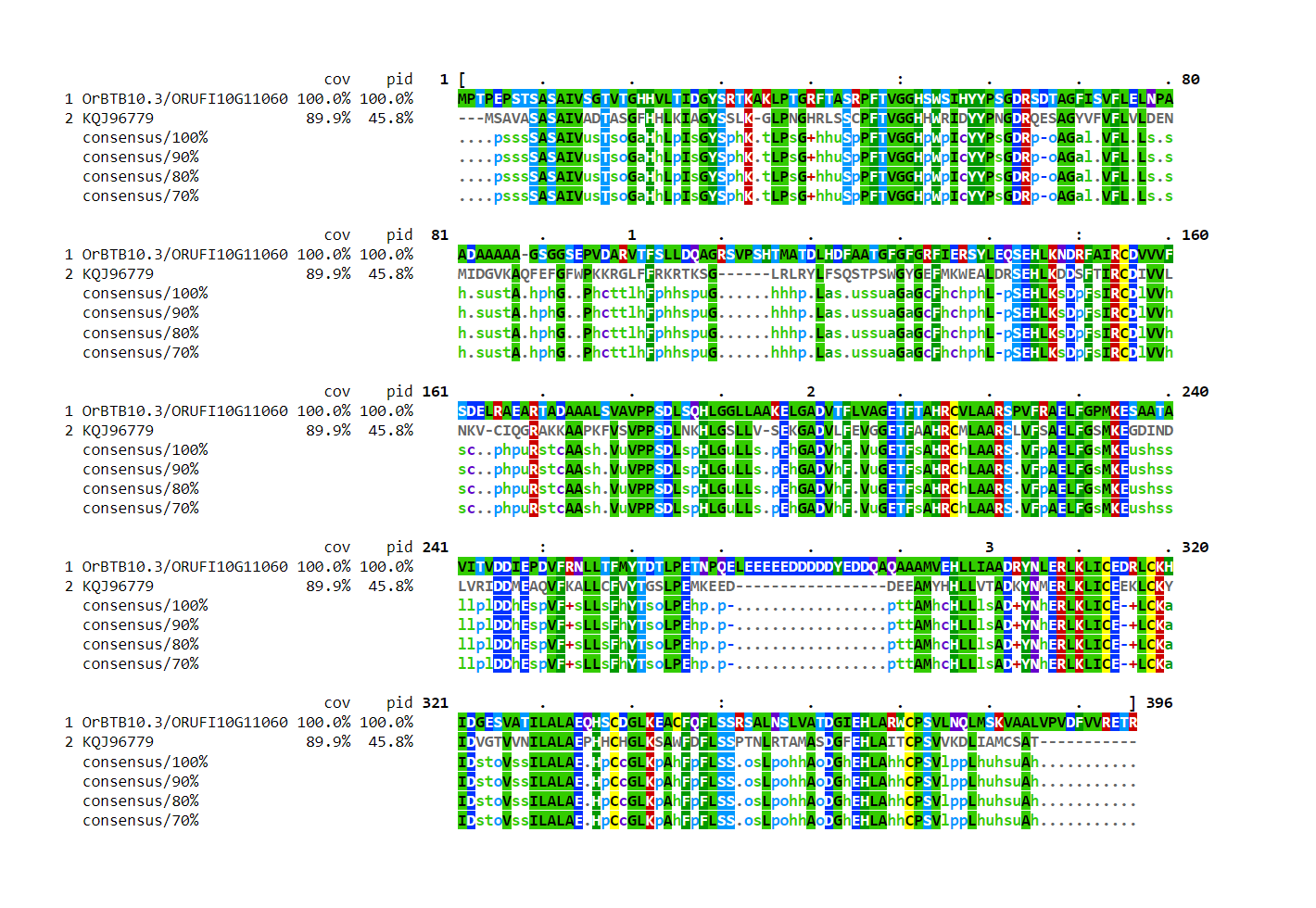


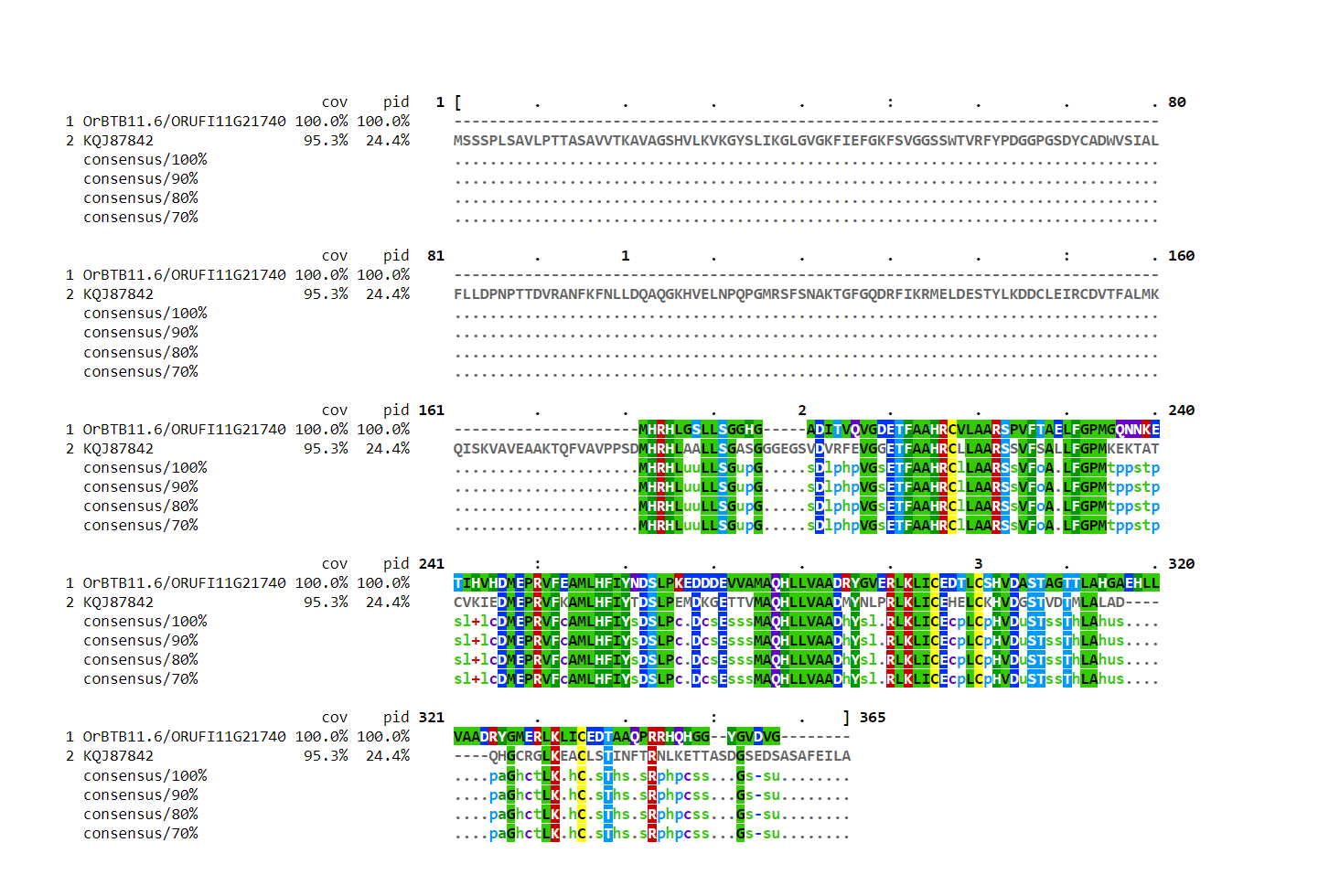


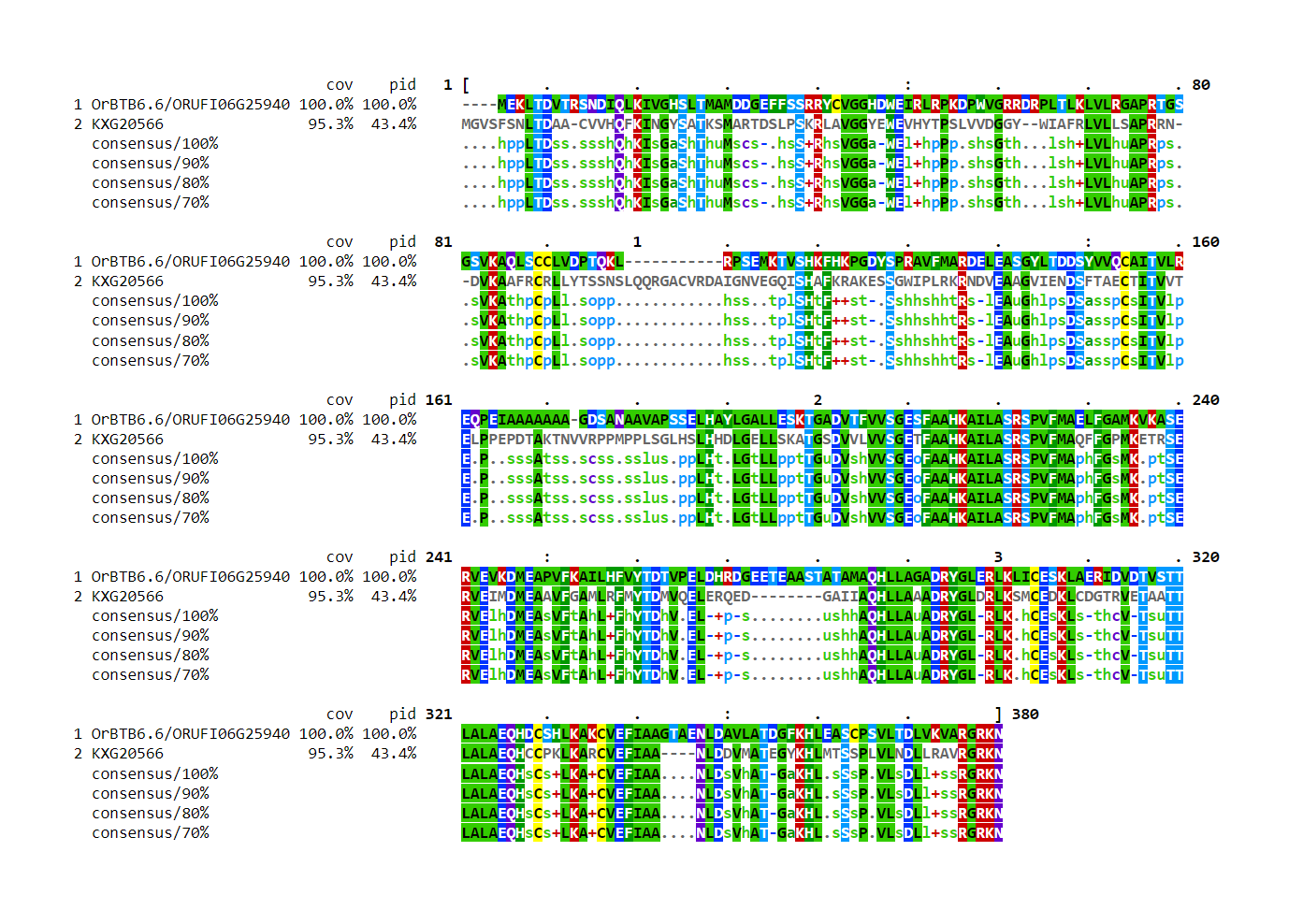


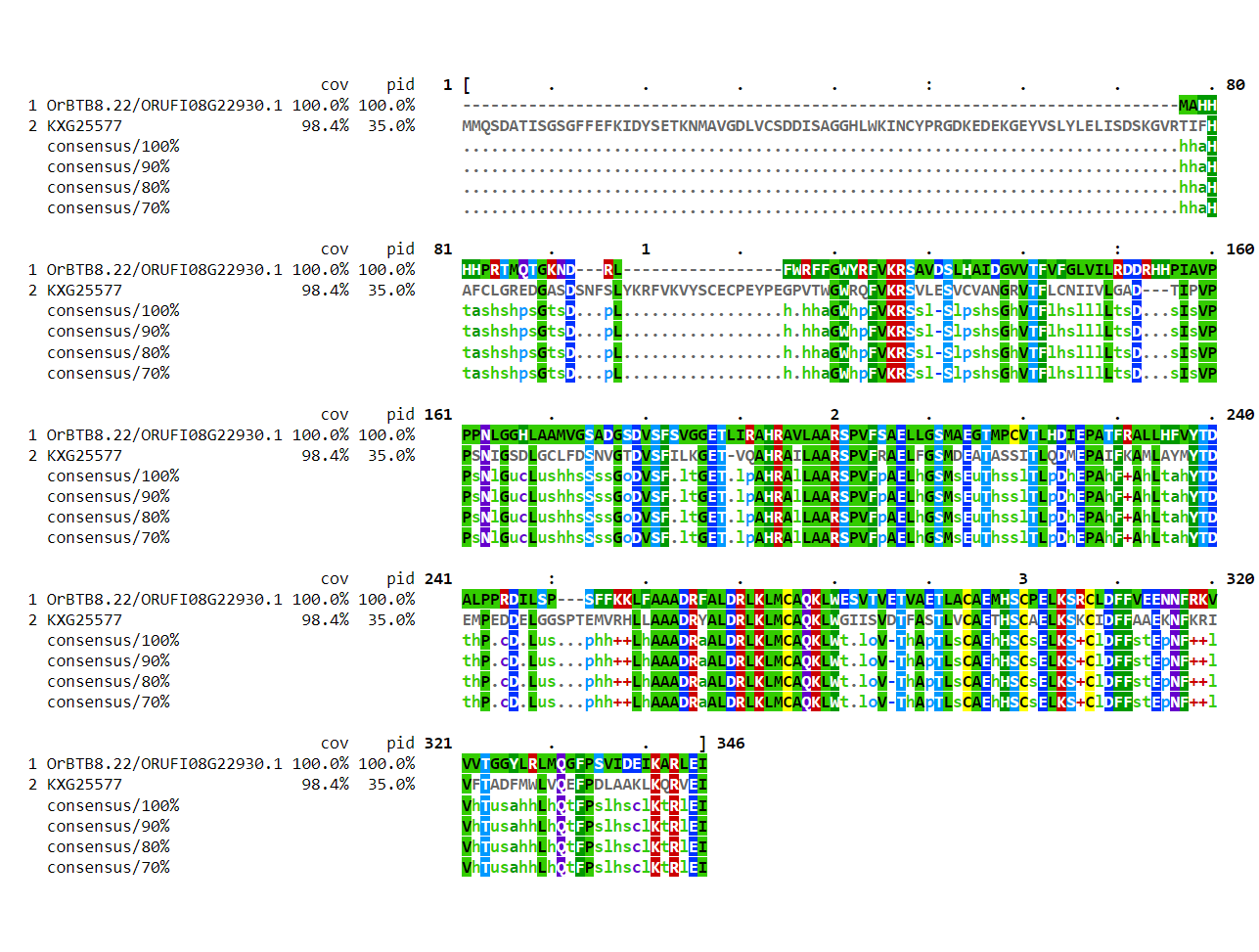


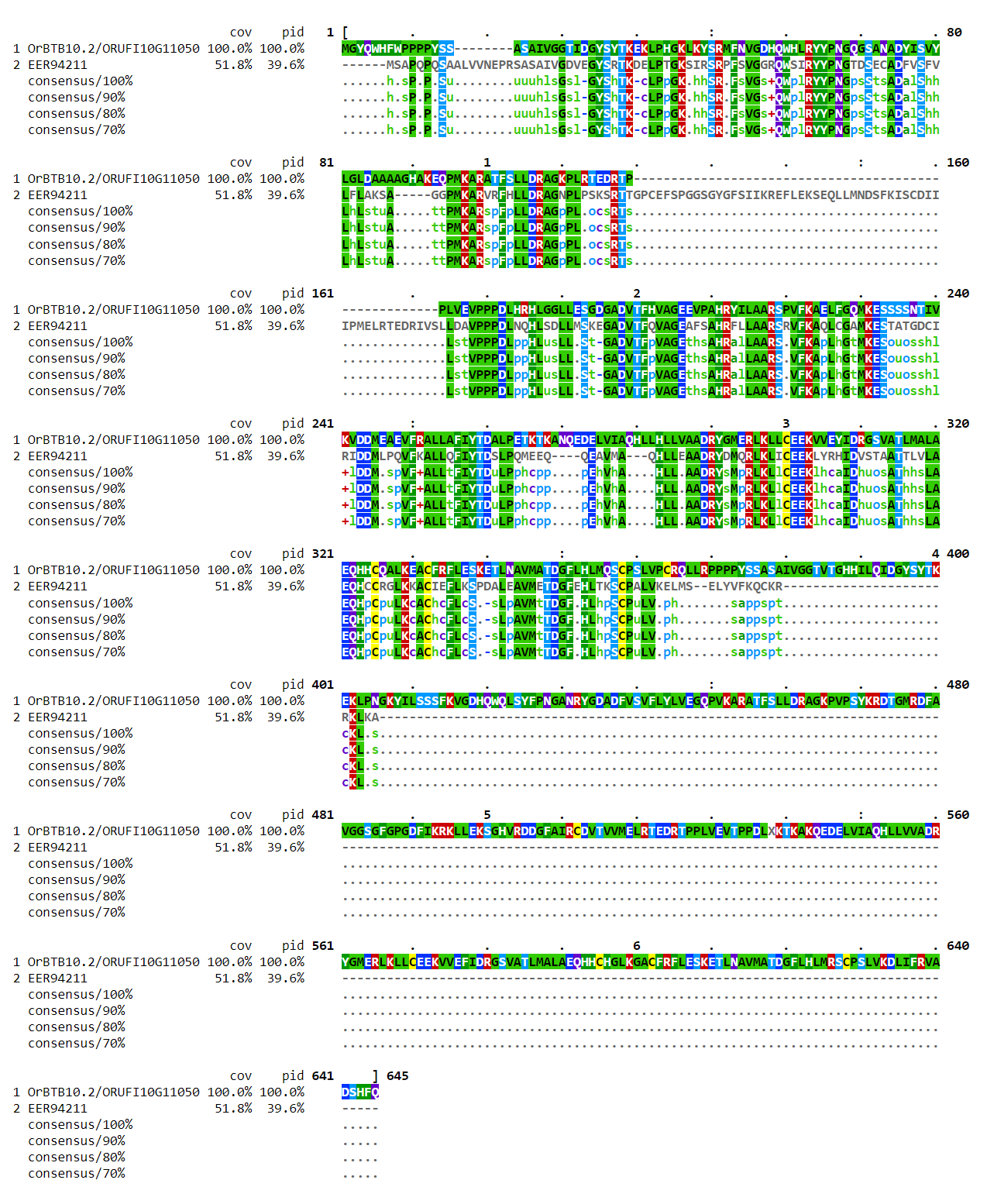


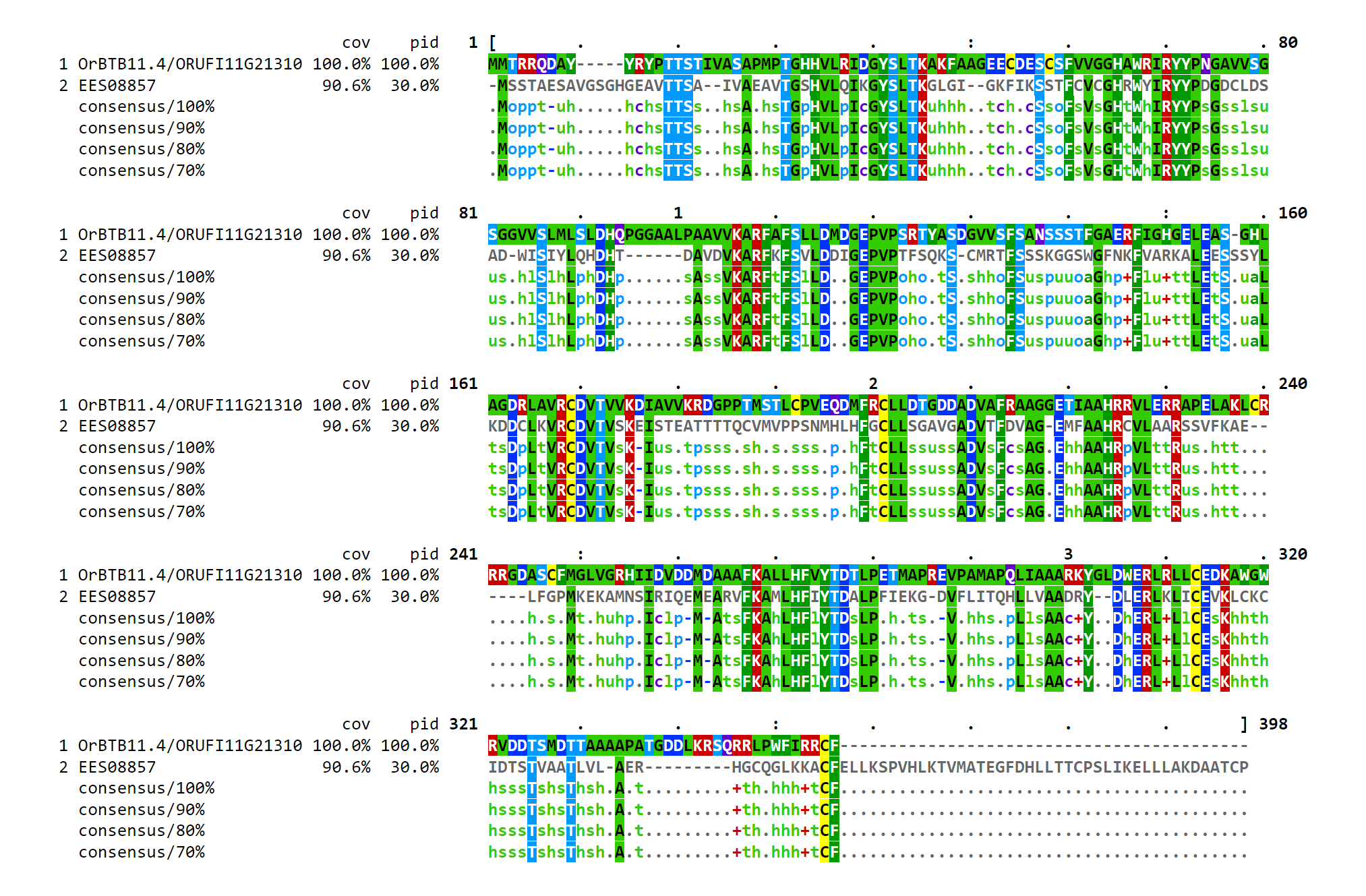


Supplementary FIGURE 5. Multiple sequence alignment of subgroup *Oryza rufipogon* BTB/POZ genes with their orthologous from *Brachypodium distachyon* and *Sorghum bicolor*. Coverage and percentage identity values are indicated in the “cov” and “pid” columns, respectively.
