# Supplementary figures and images for: Novel genes and alleles of the BTB/POZ protein family in Oryza rufipogon
Source: Sci Rep. 2023 Sep 19;13:15466. doi: 10.1038/s41598-023-41269-0 (PMC10509276; doi:10.1038/s41598-023-41269-0)

## Slide 1
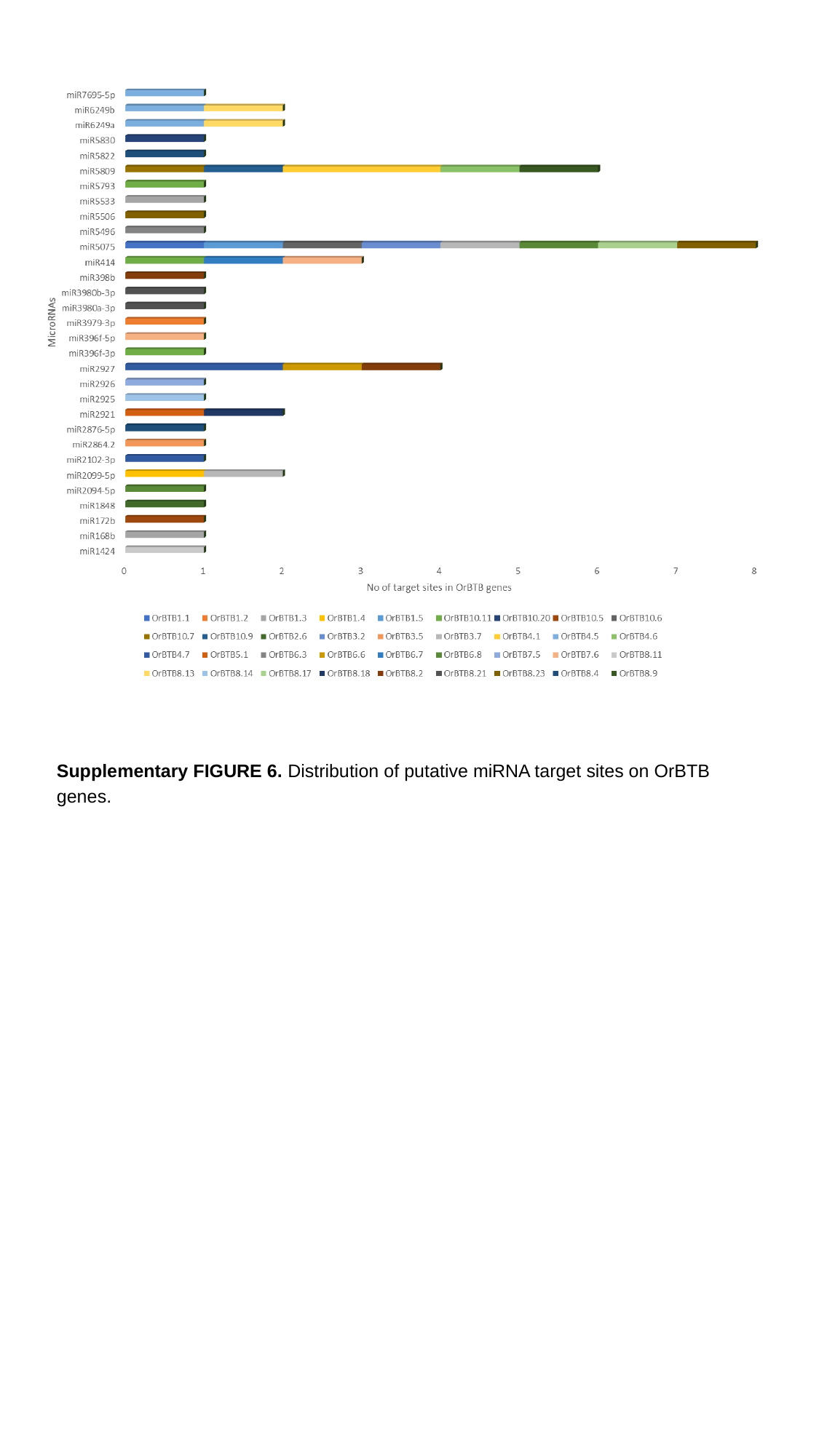

Supplementary FIGURE 6. Distribution of putative miRNA target sites on OrBTB genes.

Supplement: Supplementary file 6 — Supplementary Figure 6. [file 41598_2023_41269_MOESM6_ESM.pptx]

## Slide 1
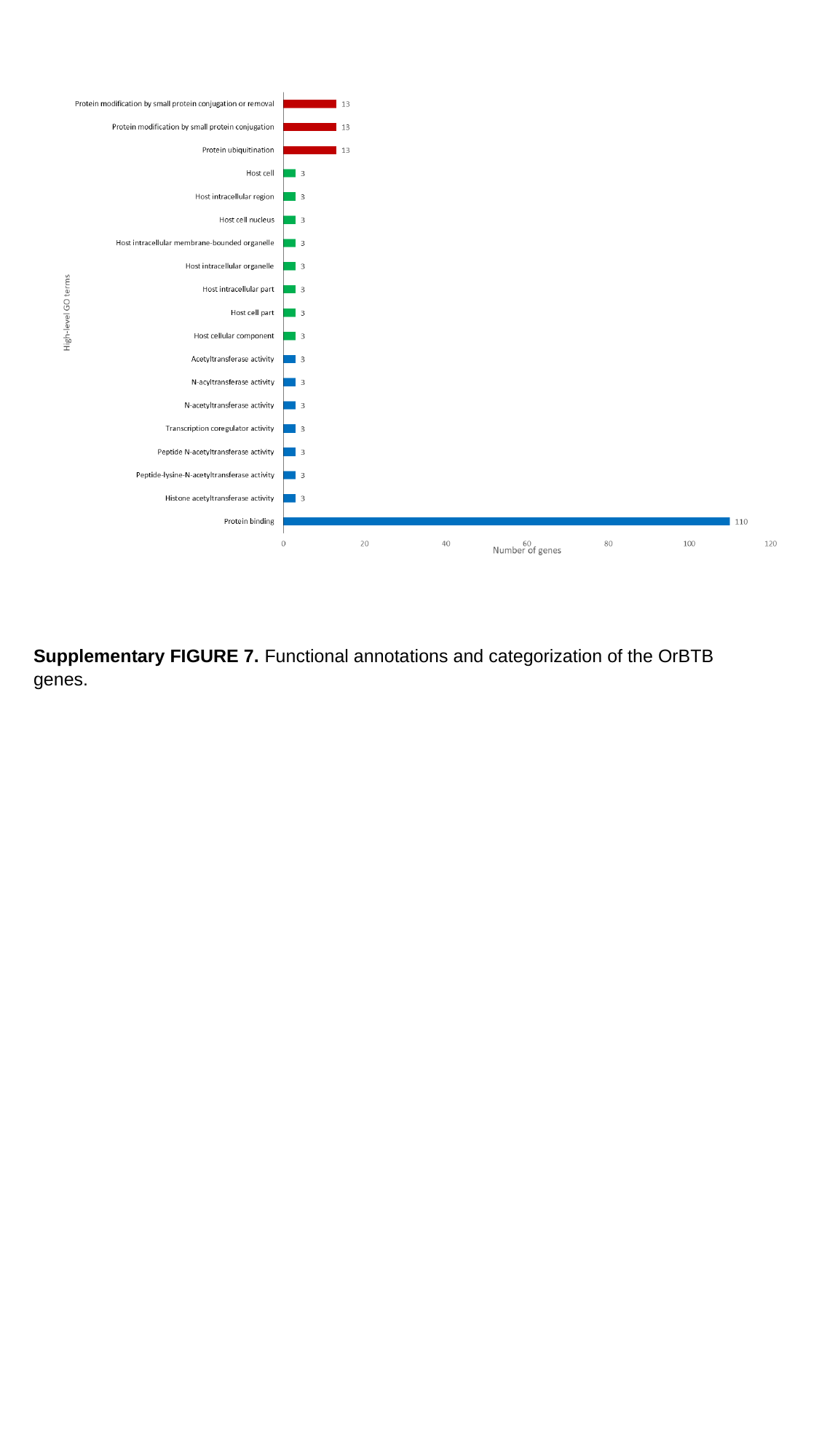

Supplementary FIGURE 7. Functional annotations and categorization of the OrBTB genes.

Supplement: Supplementary file 7 — Supplementary Figure 7. [file 41598_2023_41269_MOESM7_ESM.pptx]
